# **Simulated** microgravity induces cerebral dysfunction by disturbing protective microbiota-metabolite-microglia signaling across the gut‒brain ax**is**

**DOI:** 10.1080/19490976.2026.2635820

**Published:** 2026-02-23

**Authors:** Biying Zhang, Yue Si, Yiteng Liu, Jingjing Wei, Mengyun Li, Dailing Si, Huaxian Li, Xichen Wang, Peijun Han, Wenlan Wang, Junxiang Bao, Linfeng Cheng, Yingfeng Lei, Hongwei Ma, Yong Liu

**Affiliations:** a Department of Aerospace Hygiene, Department of Aerospace Medicine, The Fourth Military Medical University, Xi'an, Shaanxi, People's Republic of China; b Key Laboratory of Aerospace Medicine of Ministry of Education, The Fourth Military Medical University, Xi'an, Shaanxi, People's Republic of China; c School of Basic Medical Sciences, Xi'an Medical University, Xi'an, Shaanxi, People's Republic of China; d Department of Microbiology & Pathogen Biology, School of Basic Medical Sciences, The Fourth Military Medical University, Xi’an, Shaanxi, People's Republic of China; e Military Medical Innovation Center, The Fourth Military Medical University, Xi’an, Shaanxi, People's Republic of China; f Department of Pediatrics, Second Affiliated Hospital of the Fourth Military Medical University, Xi'an, Shaanxi, People's Republic of China; g Department of Anaesthesiology & Critical Care Medicine, Xijing Hospital, The Fourth Military Medical University, Xi’an, Shaanxi, People's Republic of China

**Keywords:** Simulated microgravity, gut microbiota, linoleic acid, neuroinflammation, microglia, STAT1

## Abstract

Long-duration spaceflight characterized by microgravity adversely affects operator proficiency postlanding, yet the mechanisms by which microgravity induces cerebral dysfunction refractory to short-term recovery among astronauts remain poorly defined. Here, we demonstrate that simulated microgravity (SMG) leads to chronic behavior disorders and cognitive deficits via a microbiota-metabolite-brain axis. Fecal microbiota transplantation (FMT) from long-term SMG-treated donor rats to recipients (*n* = 5 per group) under normal gravity (NG) induces anxiety-like behaviors and spatial working memory disturbances by impairing synaptic plasticity in the hippocampus, reproducing the phenotype of SMG-exposed rats. SMG destroys intestinal barriers and alters the gut microbiota to a proinflammatory state with an increased abundance of *Proteobacteria* but decreased production of linoleic acid (LA) and LA-derived metabolites, which is highly associated with neuroinflammation in the hippocampus. Mechanistically, LA can be taken up by the hippocampus under NG conditions, and then block inflammatory microglial activation by interacting with signal transducer and activator of transcription 1 (STAT1) and inhibiting its phosphorylation at Tyr 701 and Ser 727. However, the *Proteobacteria*, especially *Pseudomonas aeruginosa*, tend to be the dominant phylum in gut microbiota under SMG conditions and consume large amounts of LA, breaking LA-dependent immune homeostasis in the central nervous system (CNS). Dietary supplementation with LA significantly mitigated SMG-induced neuroinflammation and cognitive impairment. Taken together, our findings in SD rats models reveal a critical role for gut microbiota dysbiosis in simulated microgravity-associated encephalopathy, offering a novel strategy for LA replenishment to improve brain function during spaceflight.

## Article highlights

- Simulated microgravity-induced cognitive dysfunction is associated with gut microbiota dysbiosis.
- A sustained increase in the abundance of the phylum *Proteobacteria* contribute to the undue consumption of linoleic acid (LA), a metabolite that can be taken up by the microglia in the hippocampus and restrain neuroinflammation through inhibition of STAT1 signaling.
- Dietary supplementation with LA under simulated microgravity conditions could improve brain function by rebuilding the immune homeostasis in the hippocampus and mitigating the synaptic impairment.

## Graphical abstract

**Figure:**
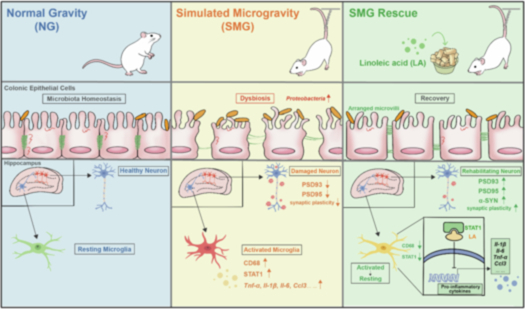

## Introduction

Spaceflight represents a frontier of human endeavor, driven by the relentless pursuit of knowledge and the expansion of our collective understanding, facilitated by rapid advancements in technology. As contemporary space missions extend in duration and frequency, astronauts are subjected to an escalating array of external stressors, including microgravity, cosmic radiation, solitude, confinement, and perturbations in circadian rhythms, which pose significant challenges to their physiological and psychological well-being.^1^ The health implications of the space environment, both in the short and long term, constitute a critical area of interest that may profoundly influence the trajectory of human space residency.

Upon entering space, astronauts are exposed to a gravitational environment markedly different from Earth's 1 G, commonly referred to as microgravity. Gravitational forces are fundamental to virtually all the physical, chemical, and biological phenomena that occur on our planet. Consequently, the abrupt absence of normal gravity (NG) and the subsequent long-term duration of microgravity lead to the loss of mechanical stimulation for cells and tissues, resulting in a cascade of deleterious effects on human health. These include, but are not limited to, a decline in cardiovascular function, muscle atrophy, osteoporosis, cerebral dysfunction, and a compromised immune system.^2–5^ The renowned *NASA Twins Study* revealed that prolonged space duration may exert a detrimental effect on operator proficiency postlanding, the cognitive deficits of which could last up to five months post-flight.^6^ The combination of mission-related stressors with the potential limitations and social isolation inherent to space travel may predispose astronauts to cognitive and psychiatric disorders.^7^ Investigating the impact of spaceflight on central nervous system (CNS) activities, including anxiety and cognitive abilities, is important for understanding future space residence.^8^ Although previous studies have indicated that microgravity, the most prominent characteristic of space life, might adversely affect neuronal activity and synaptic plasticity is response to acute stress, the specific mechanisms underlying the influence of microgravity on cerebral function remain insufficiently understood.

Microglia, the primary innate immune cells of the central nervous system, can acquire a non-canonical adaptive activation phenotype (e.g., TREM2-positive microglial activation) in chronic, low-intensity pathological microenvironments. This activation pattern exerts a mild protective effect by ameliorating tau pathology and decelerating the progression of neurodegeneration.^9^ In contrast, the prevailing consensus holds that multiple major stressors in the space environment induce neuroinflammation and microglial pathological activation, which may impair cognitive function and compromise neural homeostasis.^10^ Simulated microgravity (SMG), established by a rotating-wall vessel bioreactor for cells or the hindlimb unloading for animal models, can directly trigger pathological microglial activation and promote proinflammatory responses, synaptic pruning, and phagocytosis effects, which disrupt neuronal homeostasis.^10^ Nevertheless, how the SMG induces microglial disbalance even after short-term re-exposure to NG conditions is unclear. Recently, increasing evidence has indicated that the activation patterns of microglia are continuously and bidirectionally modulated by gut microbiota-derived metabolites. On the one hand, detrimental metabolites, such as the *Ruminococcaceae* metabolite isoamyl amine, can drive S100A8-associated proinflammatory oxidative stress responses in microglia and aggravate cognitive dysfunction.^11^ On the other hand, beneficial metabolites, such as linoleic acid (LA) from *L. acidophilus*, can activate peroxisomes in microglia, triggering microglial reprogramming to an anti-inflammatory state via reactive oxygen species (ROS) scavenging and *β*-oxidation-mediated epigenetic changes.^12^ Notably, according to the Longitudinal Study of Astronaut Health (LSAH), gastrointestinal issues rank third among medical incidents occurring during space shuttle flights.^13^ Moreover, both actual spaceflight and ground-based SMG research have revealed dysbiosis in humans and rodents, along with alterations in host metabolism.^6^^,^^14^^,^^15^ All these studies indicate that the disturbance of the gut microbiota and their metabolites caused by microgravity might affect astronaut brain function by regulating microglial activation in the long term, although the specific mechanisms have yet to be fully elucidated. This study aims to bridge this gap by investigating how microgravity alters microbiota composition and metabolite production in relation to cerebral dysfunction.

Importantly, interactions between microbiota metabolites and host receptors supporting the gut‒brain axis have been clearly demonstrated. Thus, in addition to structural changes in the brain,^16^ the gut microbiota (GM), another key factor potentially influencing the brain, is closely linked to long-duration human space missions.^17–20^ However, research on the gut‒brain axis during human spaceflight remains scarce and insufficient. Studies on International Space Station missions have shown increased *α*-diversity and decreased *β*-diversity of the human gut microbiota, with reduced populations of certain bacteria (e.g., *Akkermansia* and *Ruminococcus*).^21^ Moreover, spaceflight-induced changes in the gastrointestinal and skin microbiomes persist postflight.^21^ GM alterations are closely associated with metabolic and immunometabolic processes; for example, reduced levels of the anti-inflammatory compound 3-indolepropionic acid and increased levels of lysophosphatidylcholine may induce a proinflammatory state,^6^ potentially impacting astronaut health during long-duration space missions.

Here, we confirmed the role of the gut microbiota in SMG-associated encephalopathy through fecal microbiota transplantation (FMT) experiments and explored how the microbiota-metabolite-microglia signaling regulates cognitive deficits by integrating 16S rRNA sequencing, LC‒MS metabolomics, and microglial activation assessments. We revealed that LA, a protective metabolite hindering signal transducer and activator of transcription 1 (STAT1)-mediated inflammatory microglial activation, was excessively consumed by the *Proteobacteria* under SMG conditions. Dietary supplementation with LA could prevent cerebral dysfunction during SMG by reprogramming microglia. These findings provide a novel strategy for mitigating the brain impacts of space travel.

## Results

### Long-term SMG induces brain dysfunction by disrupting synaptic plasticity

**Figure 1.**
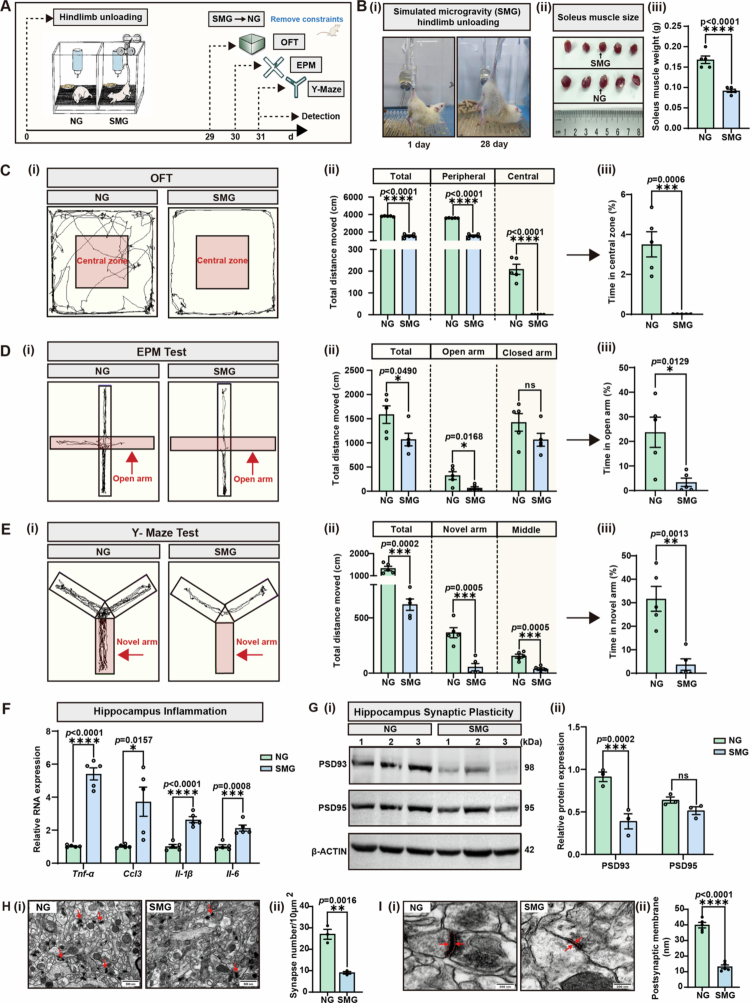
SMG induces cognitive dysfunction by impairing synaptic plasticity. **A.** The experimental design for microgravity model construction and brain function detection. After 1 week of acclimation, rats were randomly divided into two groups as follows: the normal gravity (NG, 4 weeks of horizontal tail suspension) group and the simulated microgravity (SMG, 4 weeks of hindlimb unloading) group. Behavioral tests were conducted from day 29 to day 31. **B.** (i) Schematic of hindlimb unloading model; (ii) Soleus muscle size and (iii) weight in the NG group and SMG group (*n* = 5, *p* < 0.0001). **C.** Open field test (OFT) was performed on day 29 (*n* = 5). (i) Movement trajectories of rats in OFT; (ii) Distance moved in the total path, peripheral zone, and central zone in OFT (*p* < 0.0001); (iii) Time consumption in the central zone (*p* = 0.006). **D.** Elevated plus-maze (EPM) test was performed on day 30 (*n* = 5). (i) Movement trajectories of rats in EPM; (ii) Distance moved in total path (*p* = 0.0490), open arm (*p* = 0.0168) and closed arm (ns); (iii) Time consumption in open arm (*p* = 0.0129). **E.** Y-maze (*n* = 5) test was performed on day 31. (i) Movement trajectories of rats in Y-maze; (ii) Distance moved in total path (*p* = 0.0002), novel arm (*p* = 0.0005) and middle zone (*p* = 0.0005); (iii) Time consumption in novel arm (*p* = 0.0013). **F.** qRT-PCR analysis of inflammation-related cytokines in the hippocampus was performed on day 31 (*n* = 5). *Tnf-α*, *p* < 0.0001; *Ccl3, p* = 0.0157; *Il-1β*, *p* < 0.0001; *Il-6*, *p* = 0.0008. Target gene mRNA is normalized to *β-actin* mRNA. **G.** (i) Immunoblot analysis of three independent individuals for PSD93/95 in the hippocampus. (ii) Relative protein expression of PSD93/95 by grayscale analysis. PSD93, *p* = 0.0002; PSD95, ns. **H.** SMG‐induced damage of synapse ultrastructure of the NG group and the SMG group. (i) Transmission electron microscopy (TEM) of synapses in the hippocampus (red arrow, synaptic bouton; scale bars, 500 nm). (ii) Statistics on the number of synapses, *p* = 0.0016. **I.** SMG‐induced damage of synapse ultrastructure of the NG group and the SMG group. (i) TEM of the synaptic postsynaptic membrane in the hippocampus (red arrow, synaptic postsynaptic membrane; scale bars, 200 nm). (ii) Statistics on the thickness of the postsynaptic membrane, *p* < 0.0001. Results are based on three independent biological replicates. Data are shown as the mean ± SD. The analysis is performed using the two-tailed unpaired Student’s *t* test. *p < 0.05, **p < 0.01, ***p < 0.001, ****p < 0.0001; ns no significance. Molecular weight markers are shown to the right of the blots in kDa, and target proteins are indicated to the left.

To determine whether long-term microgravity affects brain function, we established a 28-day hindlimb unweighting model to mimic the microgravity conditions in rats and detected alterations in behavioral after constraint release to mimic the postlanding circumstances. To minimize the influence of confounding factors on brain function, we simultaneously used the horizontal tail suspension in the normal gravity control group (NG group) (Figure 1A). During the modeling period, rats were able to move freely to access food and water, and maintain normal growth and health (Figure 1B-i). Compared with those in the NG group, significant muscle atrophy and weight loss of the soleus muscle were observed in the SMG group (Figure 1B-ii, iii). Multiple behavioral paradigms were subsequently used to measure the behavioral changes and cognitive performance of the rats after the hindlimb was unloaded. First, the open field test (OFT), which was used to evaluate spontaneous activity and exploratory behavior, indicated that the SMG impaired the motor ability of the rats and reduced the total distance traveled, including movement distances in the central and peripheral zones (Figure 1C-i, ii). To minimize the confounding effect of constraint on rats, the percentage of time spent in the central zone was analyzed, and the results suggested that SMG might lead to anxious-like behavior with decreased residence in the center (Figure 1C-iii). Next, the elevated plus-maze (EPM) test was applied to directly evaluate the anxiety state of the rats, and the results revealed that SMG significantly reduced the percentage of time spent in the open arms, as well as the distances of total and open arm movements (Figure 1D). The results of the Y-maze test, which was used to measure spatial learning and memory ability, demonstrated that SMGs might impair cognitive function in rats by reducing the percentage of time spent in the novel arm and decreasing the total, novel arm, and middle zone distances (Figure 1E). Collectively, these behavioral results indicated that SMG might cause encephalopathy that was characterized by anxiety and cognitive deficits in rats.

To determine how SMG affects behavior in rats, the neuropathologic signatures of the hippocampus were investigated based on previous researches.^22–24^ The transcription levels of tumor necrosis factor-*α* (*Tnf-α*), interleukin-6 (*Il-6*), interleukin-1β (*Il-1β*), and chemokine (C-C motif) ligand 3 (*Ccl3*) were significantly upregulated in the SMG group, confirming the activation of hippocampal neuroinflammation by SMG (Figure 1F). To identify changes in hippocampal synaptic plasticity caused by the SMG, immunoblot analysis was performed to measure the expression of postsynaptic density protein 93 (PSD93) and postsynaptic density protein 95 (PSD95). The results indicated that SMG disrupted hippocampal function by inhibiting synaptic protein expression (Figure 1G). Transmission electron microscopy (TEM) revealed that the synaptic structure was severely impaired in the SMG group, as indicated by significant decreases in synaptic density (Figure 1H) and postsynaptic membrane thickness (Figure 1I).

To investigate whether microglial activation contributes to SMG-induced brain dysfunction, we depleted microglia via intracerebroventricular injection of clodronate liposomes (CL). Following microglial ablation, the rats underwent 28-day hindlimb unloading to establish SMG conditions and were then subjected to behavioral tests (Figure S1A). CL treatment effectively reduced Iba-1⁺ microglial density and suppressed SMG-induced microglial proliferation, with a microglial depletion efficiency of 68.6% (Figure S1B-i, ii); notably, it also improved locomotor activity in the OFT and EPM test (Figure S1C-i, ii) and increased open arm exploration in the EPM test (Figure S1D-i, ii). These results indicate that inflammatory microglia participate in SMG-induced pathology and that their early depletion alleviates associated cognitive deficits.

In conclusion, SMG induced cerebral dysfunction by promoting neuroinflammation and impairing neuroplasticity in the hippocampus, and moreover, these pathological effects persisted even after the state was reversed to NG. How long-term SMG impair learning ability and prevent the recovery of brain function during the switch from the SMG to the NG remains ambiguous.

### Long-term of SMG impairs the intestinal barrier and affects the diversity of gut microbiota, the adverse state of which could not be restored in time even under NG conditions

**Figure 2.**
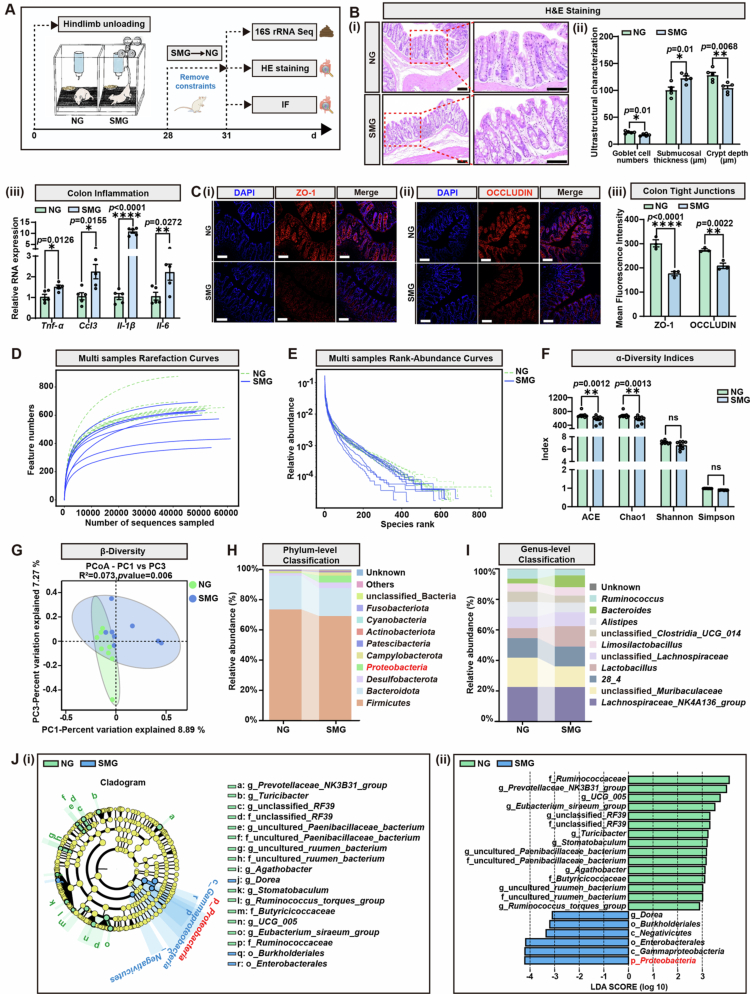
SMG triggers *Proteobacteria* expansion, intestinal barrier dysfunction, and microbiota dysbiosis. **A.** Experimental design for microgravity model construction and intestinal index detection. After 4 weeks of hindlimb unloading, fecal and tissue samples from the NG and SMG groups were collected, followed by 16S rRNA sequencing and detection. **B.** (i) Hematoxylin-Eosin (H&E) staining images of colon tissues (scale bars, 100 μm). (ii) Statistics of colonic injury characteristics in the NG and SMG groups (*n* = 5): goblet cell numbers (*p* = 0.01), submucosal thickness (*p* = 0.01), and crypt depth (*p* = 0.0068). (iii) qRT-PCR analysis of inflammation-related cytokines in the colon (*n* = 5). *Tnf-α*, *p* = 0.006; *Ccl3, p* = 0.006; *Il-1β*, *p* < 0.0001; *Il-6*, *p* = 0.0272. Target gene mRNA is normalized to *β-actin* mRNA. **C.** (i) Immunofluorescent analysis for ZO-1 localization in the colon (scale bars, 100 μm). (ii) Immunofluorescent analysis for OCCLUDIN localization in the colon (scale bars, 100 μm). (iii) Statistics on the mean fluorescence intensity; ZO-1, *p* < 0.0001; OCCLUDIN, *p* = 0.0022. **D.** Multi samples rarefaction curves of 16S rRNA sequencing (*n* = 8). **E.** Multi samples rank-abundance curves of 16S rRNA sequencing (*n* = 8). **F.** *α*-diversity shown by ACE (*p* = 0.0012), Chao1 (*p* = 0.0013), Shannon (ns), and Simpson (ns) indices (*n* = 8). **G.** *β*-diversity shown by PCoA (Bray-Curtis distance, *n* = 8). **H.** Averaged relative abundance of bacteria at the phylum level. **I.** Averaged relative abundance of bacteria at the genus level. **J.** (i) Cladogram. (ii) Linear discriminative analysis (LDA) score of differentially enriched bacterial genera obtained from LEfSe analysis between the NG group and the SMG group. A genus with Kruskal-Wallis ≤ 0.05, as well as LDA ≥ 2, is shown. Results are based on three independent biological replicates. Data are shown as the mean ± SD. The analysis is performed using two-tailed unpaired Student’s *t* test. *p < 0.05, **p < 0.01, ***p < 0.001, ****p < 0.0001; ns no significance.

It has been reported that gut microbial dysbiosis can exacerbate long-term cognitive impairments in multiple chronic CNS diseases.^25^^,^^26^ Given that microgravity disrupted intestinal mucosal homeostasis in the context of mechanical stress deficiency,^27^^,^^28^ we speculated that dysbiosis might be linked to cerebral dysfunction. To characterize the effects of microgravity on the microbiota, alterations in mucosal integrity and bacterial flora were detected based on our established SMG rat model (Figure 1). On Day 31 (3 days post-SMG treatment), intestinal and fecal samples were collected from the NG- or SMG-pretreated group for further histological and microbiome analysis (Figure 2A). Mild colitis was found in the mid-colon tissue of the SMG-pretreated rats, the HE staining features of which included moderate mucosal inflammatory cell infiltration with intact epithelium and loss of submucosal structure, but no ulceration (Figure 2B-i). Statistical analysis revealed that SMG compromised the structural integrity of the colon by significantly reducing goblet cell numbers, widening the submucosal thickness, and shortening the crypt depth (Figure 2B-ii). In fact, the pathological alterations in other tissues could also be detected. The hepatic tissues exhibited degenerative edema, blurred hepatocyte boundaries, and a disorganized lobular architecture, and the lung tissues exhibited alveolar wall thickening and collapse with a small amount of inflammatory lymphocyte infiltration (Figure S2). These findings were consistent with the previously reported pathogenesis in the hindlimb unloading-induced SMG model, providing a solid foundation for subsequent analysis.^29^^,^^30^ The transcription levels of *Tnf-α*, *Il-6*, *Il-1β*, and *Ccl3* were significantly upregulated, confirming that SMG induced continuous colonic inflammation (Figure 2B-iii). Additionally, pretreatment with SMG significantly reduced the expression of both zona occludens 1 (ZO-1) (Figure 2C-i, iii) and OCCLUDIN (Figure 2C-ii, iii), which are key tight junction proteins that maintain cell polarity, intestinal epithelial barrier integrity, and facilitate the repair of intestinal epithelial damage.^31^ These results confirmed that pre-treatment of long-term SMG pretreatment caused colonic damage.

To explore how the gut microbiome changed after long-term SMG treatment, 16S rRNA sequencing of the intestinal microbiota was conducted as designed in Figure 2A. Rarefaction curves plateaued to confirm that the data have adequate sequencing depth or coverage. Compared with the SMG group, the NG group maintained higher feature counts, indicating greater species richness in the former group (Figure 2D). Rank-abundance curves revealed that SMG stress reduced relative species abundance, distribution evenness, and diversity (Figure 2E). With respect to *α*-diversity analysis, reduced ACE/Chao1 indices resulted in a decrease in gut microbiota richness and evenness in the SMG-pretreated group (Figure 2F), and the *β*-diversity principal coordinates analysis (PCoA, Bray-Curtis distance) revealed clear clustering separation between the two groups along with the principal components explaining 16.16% of the total variance (PERMANOVA, R² = 0.073, *p* = 0.006; Figure 2G), indicating that SMG pretreatment induced systematic compositional shifts. Taxonomic profiling further uncovered functional alterations; that is, at the phylum level, SMG depleted beneficial commensals (*Firmicutes* and *Bacteroidetes*) and enriched proinflammatory taxa (*Desulfobacterota* and *Proteobacteria*; Figure 2H), a pattern mirrored at the genus level by reducing the abundance of anti-inflammatory *Alistipes/Ruminococcus* and increasing the abundance of proinflammatory *Proteobacteria* (Figure 2I). LEfSe analysis formally identified *Proteobacteria* as a signature of enriched phylum post-SMG (LDA ≥ 2, *p* < 0.05; Figure 2J), directly linking microbial dysbiosis to the inflammatory phenotype observed earlier. These findings showed that long-term SMG could cause intestinal dysfunction characterized by barrier destruction and gut dysbiosis characterized by the expansion of proinflammatory Proteobacteria, the unbeneficial state of which could not be restored quickly postlanding.

### FMT under NG conditions reproduces the behavior and cognitive phenotype of SMG

**Figure 3.**
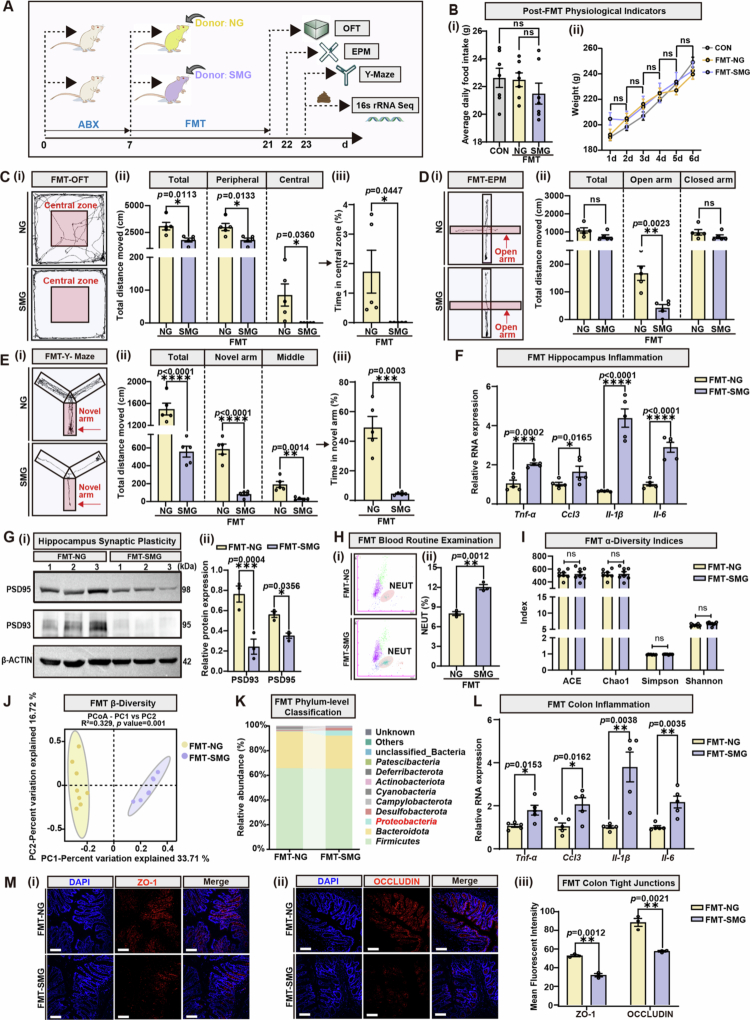
FMT-SMG induces cerebral dysfunction similar to SMG by promoting inflammation and triggering gut dysbiosis. **A.** Experimental scheme depicting fecal microbiome transplantation (FMT) from NG or SMG pre-treated rat donors to recipients. Behavioral tests were conducted on experimental days 21, 22, or 23. **B.** The physiological indicators of CONN or FMT-NG/SMG recipient rats post FMT (*n* = 5). (i) Average daily food intake from day 1 to day 6 post-FMT; (ii) Weight alteration from day 1 to day 6 post-FMT. **C.** Open field test (OFT) performed on day 21 (*n* = 5). (i) Movement trajectories of rats in OFT; (ii) Distance moved in total (*p* = 0.0113), peripheral zone (*p* = 0.0133) and central zone (*p* = 0.036) in OFT; (iii) Time consumption in central zone; *p* = 0.0447. **D.** Elevated plus-maze (EPM) test was performed on day 22 (*n* = 5). (i) Movement trajectories of rats in EPM; (ii) Distance moved in the total path (ns), open arm (*p* = 0.0023), and closed arm (ns). **E.** Y-maze test performed on day 23 (*n* = 5). (i) Movement trajectories of rats in Y-maze; (ii) Distance moved in the total path (*p* < 0.0001), novel arm (*p* < 0.0001), and middle arm (*p* = 0.0014); (iii) Time consumption in the novel arm (*p* = 0.0003). **F.** qRT-PCR analysis of inflammation-related cytokines in the hippocampus (*n* = 5). *Tnf-α*, *p* = 0.0002; *Ccl3*, *p* = 0.0165; *Il-1β*, *p* < 0.0001; *Il-6*, *p* < 0.0001. Target gene mRNA is normalized to *β-actin* mRNA. **G.** (i) Immunoblot analysis of three independent individuals for PSD93/95 in the hippocampus. (ii) Relative protein expression of PSD93 (*p* = 0.0004) and PSD95 (*p* = 0.0356) by grayscale analysis. **H.** The percentage of neutrophils in blood was detected by flow cytometry assays (*p* = 0.0012). **I.** *α*-diversity shown by ACE (ns), Chao1 (ns), Shannon (ns), and Simpson (ns) indices (*n* = 8). **J.** *β*-diversity shown by PCoA (*n* = 8). **K.** Averaged relative abundance of bacteria at the phylum level. **L.** qRT-PCR analysis of inflammation-related cytokines in the colon (*n* = 5). *Tnf-α*, *p* = 0.0153; *Ccl3*, *p* = 0.0162; *Il-1β*, *p* = 0.0038; *Il-6*, *p* = 0.0035. Target gene mRNA is normalized to *β-actin* mRNA. **M.** (i) Immunofluorescent analysis for ZO-1 localization in colon (scale bars, 100 μm); (ii) Immunofluorescent analysis for OCCLUDIN localization in colon (scale bars, 100 μm); (iii) Statistics on the mean fluorescence intensity of (i-ii); ZO-1, *p* = 0.0012; OCCLUDIN, *p* = 0.0021. Results are based on three independent biological replicates. Data are shown as the mean ± SD. The analysis is performed using two-tailed unpaired Student’s *t* test. *p < 0.05, **p < 0.01, ***p < 0.001, ****p < 0.0001; ns no significance.

To determine the role of the gut microbiota in microgravity-induced brain dysfunction, feces from the NG- or SMG-treated rats were transplanted into recipients under NG conditions via a 14-day FMT protocol (FMT-NG group vs FMT-SMG group) (Figure 3A). No significant differences in average daily food intake or body weight gain were detected between the two groups and the control group (CONN) (Figure 3B), ruling out the interference of FMT on the general health status of the rats. Multidimensional behavioral paradigms were subsequently employed to evaluate brain functional changes in the brain. The OFT results revealed that compared with the FMT-NG group, the FMT-SMG group exhibited shorter total, central, and peripheral zone movements and a marked reduction in the percentage of time spent in the central zone (Figure 3C). The results of EPM test revealed that FMT-SMG rats maintained decreased movements in the open arm, whereas the total distance traveled remained unchanged (Figure 3D). The results of the Y-maze test suggested that the FMT-SMG rats displayed shorter total movements or movements in the novel arm and middle zone, with a decreased percentage of time spent in the novel arm (Figure 3E). These behavioral abnormalities were highly consistent with findings from the previously established SMG model (Figure 1), collectively indicating that FMT recapitulates SMG-induced anxiety-like behaviors and mild cognitive impairment.

To investigate how FMT from SMG-treated rats affects brain function, the levels of neuro- and systemic inflammatory indicators, as well as alterations in the gut microbiome and mucosal barrier, were detected. qRT-PCR analysis revealed significantly upregulated transcription of proinflammatory cytokines, such as *Tnf-α*, *Il-6*, *Il-1β*, and the chemokine *Ccl3*, in the hippocampus of the FMT-SMG group (Figure 3F), indicating that excessive activation of neuroinflammation could be triggered by FMT. Immunoblot analysis further revealed that reduced expression of PSD93 and PSD95 in the FMT-SMG group compared with the FMT-NG group (Figure 3G), suggesting that exacerbated neuroinflammation caused by SMG-associated gut dysbiosis might impair the hippocampal synaptic plasticity. Additionally, the peripheral inflammatory status was also assessed. The blood routine examination demonstrated a significant increase in neutrophil percentage in the FMT-SMG group (Figure 3H), indicating activated systemic inflammatory responses. To further elucidate the role of the gut microbiota in SMG-induced behavior and the cognitive phenotype, 16S rRNA sequencing was performed to characterize alterations in the gut microbiota of FMT recipient rats. Sequencing revealed that although there were no significant changes in the *α*-diversity indices (Figure 3I), the rarefaction curves and rank-abundance curves indicated an overall decreasing trend in the richness and evenness of the microbiota in the FMT-SMG group (Figures S3A-B). *β*-diversity analysis via PCoA based on Bray-Curtis distances revealed distinct clustering of microbial communities between the FMT-NG and FMT-SMG groups along the PC1 (explaining 33.71% of the variance) and PC2 (16.72% of variance) axes (R² = 0.329, *p* = 0.001) (Figure 3J), confirming the stable transmission of SMG-associated microbiota profiles through FMT. In-depth taxonomic profiling at the phylum level, combined with linear discriminant analysis (LDA) effect size (LEfSe) differential testing (LDA ≥ 2, *p* < 0.05), revealed significant enrichment of *Desulfobacterota* and *Verrucomicrobiota* in the FMT-SMG group, accompanied by the expansion of *Proteobacteria*, whereas *Patescibacteria* and c*yanobacteria* were dominant in the FMT-NG group (Figure 3K, Figure S3C). Kyoto Encyclopedia of Genes and Genomes (KEGG) pathway analysis revealed that dominant bacteria in the FMT-SMG group were significantly enriched in pathways such as lipid metabolism, nucleotide metabolism, and metabolism of other amino acids (Figure S3D). These findings indicated that FMT-SMG could induce microbiota dysbiosis patterns similar to those observed in the original SMG model. Considering the intimate crosstalk between alterations in the gut microbiota and intestinal mucosa impairment, the pathological data of the colonic tissue were further collected and evaluated with different indicators. The qRT-PCR results revealed upregulated transcription of proinflammatory factors *Tnf-α*, *Il-6*, *Il-1β*, and *Ccl3* in FMT-SMG colons (Figure 3L), signifying that the local intestinal inflammation was enhanced by the FMT-SMG treatment. Immunofluorescence staining revealed significantly decreased expression of the tight junction proteins ZO-1 and OCCLUDIN in FMT-SMG intestines (Figure 3M), confirming that FMT-SMG treatment could contribute to aggravated mucosal barrier damage and increased epithelial permeability. These intestinal pathological changes most likely formed a vicious cycle with microbiota dysbiosis, collectively mediating SMG-related gut–brain axis dysfunction.

In summary, FMT of the SMG-associated microbiota recapitulates SMG-associated behavioral abnormalities through the simultaneous induction of intestinal, hippocampal, and systemic inflammation, as well as mucosal barrier injury and synaptic plasticity deficits. These findings confirmed that gut microbiota dysbiosis may be a critical mediator of the pathogenesis of long-term SMG-induced cerebral dysfunction.

### The proinflammatory *Proteobacteria* accelerate the consumption of protective linoleic acid (LA) under SMG conditions

**Figure 4.**
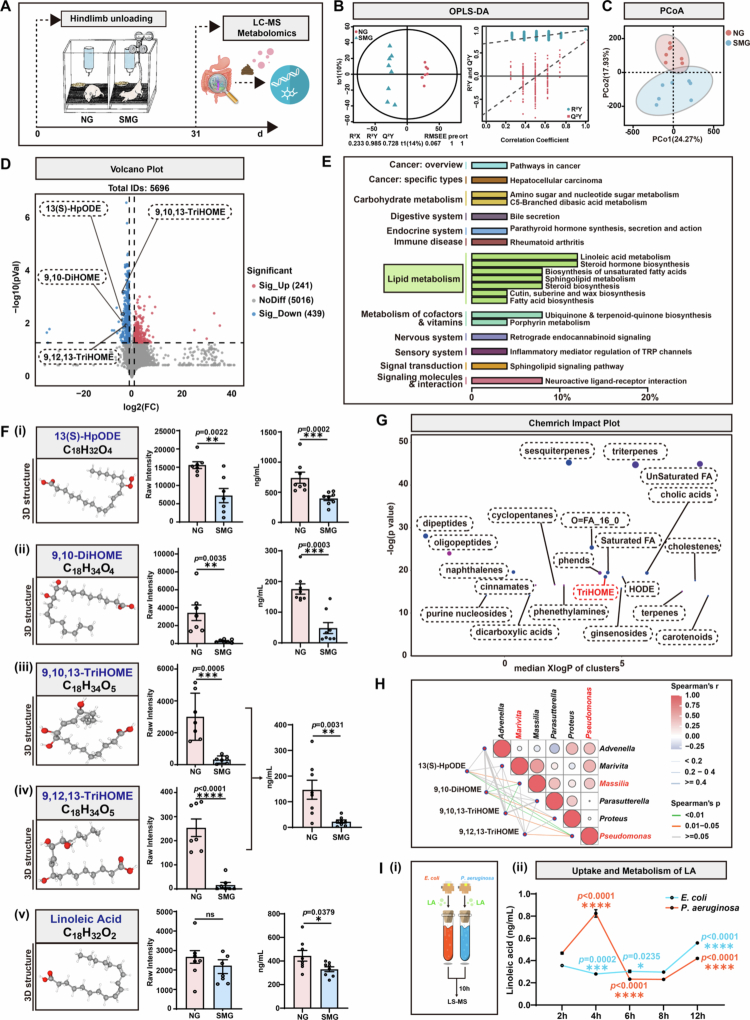
Protective LA is consumed at an accelerated rate by proinflammatory*Proteobacteria*under SMG conditions. **A.** After 28 days of hindlimb unloading (SMG model), feces were collected from the NG or SMG group for liquid chromatography-mass spectrometry (LC-MS) metabolomics on day 31. **B.** Changes in gut microbial metabolites (orthogonal projections to latent structures - discriminant analysis, OPLS-DA). **C.** Principal coordinates analysis (PCoA) plotting of gut microbial metabolites of NG and SMG. **D.** Differentially expressed metabolites were identified by volcano plots. **E.** Kyoto Encyclopedia of Genes and Genomes (KEGG) analysis evaluated the enriched pathways (lipid metabolism) for the representative profiles of NG and SMG groups. **F.** The raw intensity and concentration of LA and its derivatives. (i) 13(S)-HpODE, *p* = 0.0022/*p* = 0.0002; (ii) 9, 10-DiHOME, *p* = 0.0035/*p* = 0.0003; (iii) 9, 10, 13-TriHOME, *p* = 0.0005/*p* = 0.0031; (iv) 9, 12, 13-TriHOME, *p* < 0.0001/*p* = 0.0031; (v) LA, ns/*p* = 0.0379. **G.** Chemrich impact plotting of gut microbial metabolites of NG and SMG. **H.** Spearman’s correlation analysis of microbiome and metabolome. **I.** Liquid chromatography-mass spectrometry (LC-MS) analysis of LA levels in *E. coli* and *P. aeruginosa.* (i) Experimental design; (ii) The concentration of LA in 2 h, 4 h, 6 h, 8 h, 10 h. Results are based on three independent biological replicates. Data are shown as the mean ± SD. The analysis is performed using two-tailed unpaired Student’s *t* test. *p < 0.05, **p < 0.01, ***p < 0.001, ****p < 0.0001; ns no significance.

Previous studies have suggested that the gut microbiota can influence the outcome of systemic inflammation through the production or consumption of metabolites. Therefore, we hypothesized that SMG-induced neuroinflammation is also associated with metabolic dysregulation. To verify this hypothesis, we performed untargeted liquid chromatography‒tandem mass spectrometry (LC‒MS/MS) metabolomics on fecal samples from NG and SMG rats (Figure 4A). Data validity was first confirmed using multivariate statistical modeling. Orthogonal partial least squares discriminant analysis (OPLS-DA) revealed excellent model fit (R²Y ≈ 1) and predictive power (Q²Y > 0.5), ensuring robust exclusion of bias in both the NG and SMG groups for subsequent differentially abundant metabolite screening (Figure 4B). Principal coordinates analysis (PCoA) based on Bray‒Curtis distances revealed significant separation of metabolic communities along principal components PC1 (24.27% variance) and PC2 (17.93% variance), with tight intragroup clustering and clear intergroup discrimination (Figure 4C). This visually confirmed that SMG-induced specific metabolic phenotype alterations, guiding targeted differential analysis. Using stringent criteria (log_2_FC > 1, FDR < 0.05), 241 upregulated and 439 downregulated metabolites were identified in the SMG group (Figure 4D), serving as key targets for identifying SMG-induced metabolic perturbations. KEGG pathway analysis revealed significant enrichment of lipid metabolism pathways, with LA metabolism and steroid hormone biosynthesis as the dominant alternated pathways (Figure 4E). LA or its derivatives have neuroprotective effects against oxidative and inflammatory injury in multiple CNS diseases.^32^^,^^33^ Given that LA derivatives, such as omega-6 polyunsaturated fatty acid metabolites, can penetrate the blood‒brain barrier and mediate neuroprotective effects,^34–36^ the LA metabolic pathway was prioritized for in-depth investigation. Targeted analysis revealed that the levels of four downstream LA derivatives, namely, 13(S)-HpODE, 9, 10-DiHOME, 9, 10, 13-TriHOME, and 9, 12, 13-TriHOME, were significantly decreased after SMG treatment (Figure 4F). Chemical structure clustering revealed that all four derivatives belonged to the TriHOME chemical family (Figure 4G). However, the LA levels showed a decreasing trend but remained unchanged post-SMG exposure, the imprecise quantification inherent to untargeted sequencing required confirmation by targeted metabolite detection, which definitively validated the downregulation of LA and its derivatives (Figure 4F). Further absolute quantitative metabolomic analysis of the hippocampus revealed that SMG exposure decreased LA and its derivatives (Figure S5), suggesting that gut microbiota-derived LA and its derivatives may regulate central metabolic homeostasis via the gut-brain axis, and their downregulation may contribute to SMG-induced cognitive impairment and neuroinflammation.

To decipher how microbiota alteration affects metabolite production, Spearman’s correlation analysis was performed to quantify the correlations between bacterial abundance and metabolite levels, with correlations adjusted for multiple comparisons using the Benjamini-Hochberg FDR method. 13(S)-HpODE, 9, 10-DiHOME and 9, 10, 13-TriHOME were negatively correlated with *Massilia*, and all four LA derivatives were negatively correlated with *Pseudomonas aeruginosa* (*P. aeruginosa*) (Figure 4H), both of which belonged to the proinflammatory *Proteobacteria* and are associated with the depletion of protective metabolites.

To address the limitations of single-genus analysis, global testing across all SMG-enriched taxa revealed eight proinflammatory species, including *Amedibacillus dolichus* and *Shigella flexneri*, which were negatively correlated with the beneficial TriHOME derivatives (Figure S4), reinforcing the pathological axis, namely, enrichment-protective metabolite depletion of proinflammatory bacteria, at the community level. Given *Escherichia coli* (*E. coli*) as a commensal *Proteobacteria*, and *P. aeruginosa* as the most significantly enriched conditional pathogen in this study, these species were cocultured with 100 μM LA for 10 hours. Prior to this assay, both strains were pre-adapted to SMG and a hypoxic environment (10% O₂) for 30 days *in vitro*. Furthermore, to ensure consistent initial biomass, all cultures were washed by centrifugation and resuspended in fresh medium to normalize the starting OD₆₀₀ to 0.1 before the addition of LA. LA content in bacteria was quantified at multiple time points via LC‒MS, and the rates of LA uptake and consumption were compared between the two groups. Compared with *E. coli*, *P. aeruginosa* exhibited rapid LA uptake during the logarithmic phase (0–4 h) and significantly greater LA consumption during the stationary phase (4–10 h) (Figure 4I). These findings suggested that in the SMG-altered intestinal microenvironment, the dominant presence of pathogenic *Proteobacteria* like *P. aeruginosa* synchronized with aberrant patterns of metabolite consumption. This was potentially suggested by a correlation between increased LA uptake by *Proteobacteria* and the depletion of TriHOME derivatives. In brief, SMG was associated with a concurrent increase in proinflammatory *Proteobacteria*, accelerated consumption of LA pathway-derived TriHOME family derivatives, and disruptions in gut–brain axis signaling.

### Oral supplementation with LA improves SMG-induced cerebral dysfunction by enhancing hippocampal synaptic plasticity

**Figure 5.**
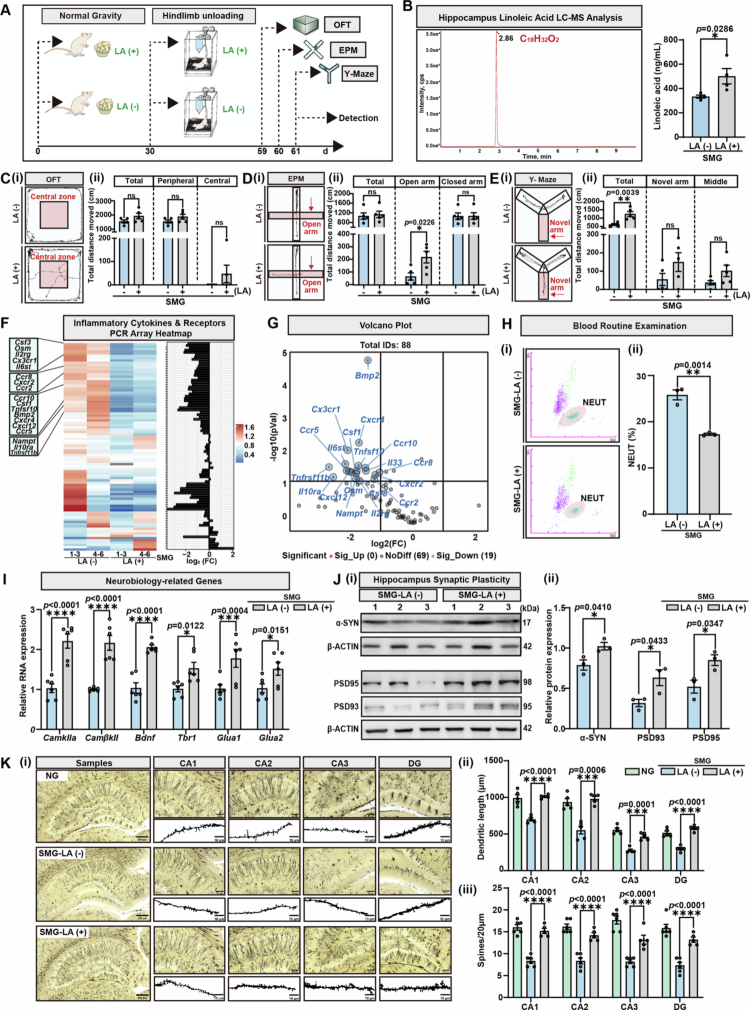
LA supplementation alleviates SMG-induced cerebral dysfunction by enhancing hippocampal synaptic plasticity. **A.** Schematic of 10% (w/w) LA treatment in SMG rats for 58 days (pretreatment for 30 days, plus hindlimb unloading for 28 days). Behavioral tests were conducted from day 59 to day 61. **B.** LC-MS analysis of LA level in hippocampus of day 61 (*n* = 4), *p* = 0.0286. **C.** Open field test (OFT) was performed on day 5 (*n* = 5). (i) Movement trajectories of rats in OFT; (ii) Distance moved in the total path (ns), peripheral zone (ns), and central zone (ns) in OFT. **D.** Elevated plus-maze (EPM) test was performed on day 60 (*n* = 5). (i) Movement trajectories of rats in EPM; (ii) Distance moved in total (ns), open arm (*p* = 0.0226), and closed arm (ns). **E.** Y-maze test was performed on day 61 (*n* = 5). (i) Movement trajectories of rats in Y-maze; (ii) Distance moved in total (*p* = 0.0039), novel arm (ns), and middle arm (ns). **F.** PCR array heatmap analysis of hippocampal inflammatory cytokines & receptors in SMG-LA (−) and SMG-LA ( + ) groups. **G.** Differentially expressed hippocampal inflammatory cytokines were identified by volcano plots. **H.** The percentage of neutrophils in blood by flow cytometry (*p* = 0.0014). **I.** qRT-PCR analysis of neurobiology-related genes in the hippocampus (*n* = 5). *CamkIIa*, *p* < 0.0001; *CamkIIβ*, *p* < 0.0001; *Bdnf*, *p* < 0.0001; *Tbr1*, *p* = 0.0122; *Glua1, p* = 0.0004; *Glua1, p* = 0.0151. Target gene mRNA is normalized to *β-actin* mRNA. **J.** (i) Immunoblot analysis of three independent individuals for *α*-syn, PSD93/95 in the hippocampus. (ii) Relative protein expression of *α*-SYN (*p* = 0.041), PSD93 (*p* = 0.0433) and PSD95 (*p* = 0.0347) by grayscale analysis. **K.** (i) Representative images of dendritic segments, scale bar = 500/10 μm. (ii) Dendritic length of hippocampal neurons. CA1, *p* < 0.0001; CA2, *p* = 0.0006; CA3, *p* = 0.0001; DG, *p* < 0.0001. (iii) Total spine density in hippocampal neurons. CA1, *p* < 0.0001; CA2, *p* < 0.0001; CA3, *p* < 0.0001; DG, *p* < 0.0001. Results are based on three independent biological replicates. Data are shown as the mean ± SD. The analysis is performed using two-tailed unpaired Student’s *t* test. *p < 0.05, **p < 0.01, ***p < 0.001, ****p < 0.0001; ns no significance.

Given that LA consumption by gut proinflammatory bacteria cooccurs with SMG-induced brain injury, oral administration of LA was performed by feeding rats a special diet containing 10% (w/w) LA for two months (Figure 5A, Figure S8-i). LC–MS confirmed that hippocampal LA levels were significantly greater in the LA-supplemented group (SMG-LA (+)) than in the control group (SMG-LA (−)) (Figure 5B), demonstrating that exogenous LA could pass through the blood–brain barrier and accumulate in the CNS. Behavioral tests revealed that while total movement distance did not differ significantly between the SMG-LA (+) and SMG-LA (−) groups, SMG-LA (+) rats exhibited increased exploration activity in the central zone of the OFT (Figure 5C), longer movement distance in the open arms of the EPM test (Figure 5D), and a trending increase in exploration activity in the novel arm of the Y-maze (Figure 5E). Simultaneously, we performed FMT using fecal matter from SMG donors, with concomitant administration of LA to the donors (Figure S6A). In the FMT‑SMGLA (+) group, increased total distance traveled and center distance were observed in the OFT, along with increased novel arm distance in the Y‑maze test (Figure S6B). Transcript levels of inflammation‑related factors were reduced in both the colon and hippocampus of the FMT‑SMGLA (+) group (Figure S6C-D). The down‑regulated synaptic plasticity proteins PSD93 and PSD95 in the hippocampus, as well as the elevated STAT1 and p‑STAT1 (Tyr 701), were restored after the supplement of LA (Figure S6E). These behavioral changes indicated that LA supplementation specifically mitigated SMG-induced anxiety-like behaviors, spatial exploration deficits, and concomitant neuroinflammation.

To investigate the neuroprotective mechanisms of LA, a PCR array for rat inflammatory cytokines and receptors was used to profile gene expression in the rat hippocampus. The result of the PCR array results revealed that SMG-LA (+) downregulated nineteen kinds of inflammatory cytokines and receptors, including one chemokine (C-X-C motif chemokine ligand 12-*Cxcl12*), seven chemokine receptors (e.g., C-X3-C motif chemokine receptor 1-*Cx3cr1*, C-X-C motif chemokine receptor 4-*Cxcr4*, C-C motif chemokine receptor 5-*Ccr5*), two interleukins (interleukin 33-*Il33*, and interleukin 2 receptor gamma chain-*Il2rg*), two interleukin receptors (interleukin 6 signal transducer-*Il6st* and interleukin 10 receptor alpha chain-*Il10ra*), six other cytokines (e.g., bone morphogenetic protein 2-*Bmp2*, colony stimulating factor 1-*Csf1*, tumor necrosis factor superfamily member 10-*Tnfsf10*), and one cytokine receptor (tumor necrosis factor receptor superfamily member 11b-*Tnfrsf11b*) (Figure 5F-G). These findings indicated a significant reduction in neuroinflammation in the CNS, revealing the anti-inflammatory effects of LA. Blood routine examination further revealed that the percentage of neutrophils was significantly lower in the SMG-LA (+) group than in the SMG-LA (−) group (Figure 5H), indicating that supplementation with LA could ameliorate SMG-induced systemic inflammation.

To elucidate the underlying mechanisms through which LA supplementation ameliorates brain function, we investigated alterations in hippocampal synaptic plasticity. qRT-PCR revealed that SMG-LA (+) promoted the expression of multiple neurotrophic factors (such as brain-derived neurotrophic factor-*Bdnf*), CaMK family (such as calcium/calmodulin-dependent protein kinase II alpha-*CamkIIα*, and calcium/calmodulin-dependent protein kinase II beta-*CamkIIβ*), AMPA (such as glutamate receptor 1-*GluA1* and glutamate receptor 2-*GluA2*) receptors, and cortical development factor (such as t-box brain protein 1-*Tbr1*) in the hippocampus (Figure 5I). Moreover, immunoblot analysis confirmed that supplementation with LA promoted the expression of *α*-synuclein (*α*-Syn), PSD93, and PSD95 in hippocampal tissues (Figure 5J), indicating that LA intervention repaired synaptic plasticity damage in the SMG environment. Golgi staining directly revealed changes in synaptic structure, the results of which revealed that LA supplementation restored the dendritic length and spine density in hippocampal subregions (CA1, CA2, CA3, and DG) (Figure 5K), providing morphological evidence of neuronal structural protection. Considering that intestinal barrier damage is critical for the early pathogenesis of SMG, TEM was performed to evaluate colon injury with or without oral LA administration. TEM revealed that the NG maintained abundant and orderly microvilli, intact columnar epithelial cells (blue arrows), complete glycocalyx (red arrows), and tight junctions (yellow arrows); however, the SMG or FMT-SMG groups exhibited fewer and more disorganized microvilli, enlarged lysosomes (green arrows), and disrupted tight junctions (Figure S7). SMG-LA (+) significantly restored intestinal epithelial structure, with increased and organized microvilli, a reappearing glycocalyx, and more intact tight junctions. These data indicated that LA supplementation could concurrently improve intestinal mucosal integrity. In conclusion, this study demonstrated that dietary LA supplementation was associated with a restoration of protective metabolites depleted by *Proteobacteria*, an alleviation of neuroinflammation, and a concomitant improvement in synaptic integrity and cerebral function under SMG conditions.

### LA administration enhances the excitability of hippocampal CA1 pyramidal neurons in rat SMG model

**Figure 6.**
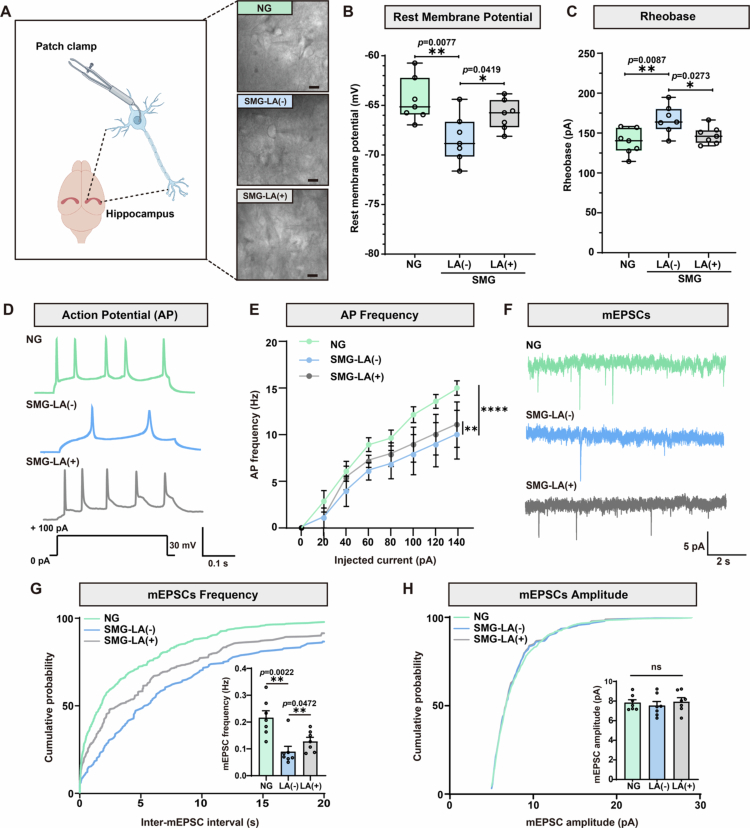
Altered electrophysiology of hippocampal CA1 pyramidal neurons after LA administration in SMG rats. **A.** Schematic diagram of the patch clamp recording in the hippocampal CA1 region and representative images (scale bars, 20 μm). **B.** Resting membrane potential of hippocampal CA1 pyramidal neurons in NG, SMG-LA (−), and SMG-LA (+) groups. **C.** Rheobase current of hippocampal CA1 pyramidal neurons in NG, SMG-LA (−), and SMG-LA (+) groups. **D.** Example traces of current-clamped neurons from NG, SMG-LA (−), and SMG-LA (+) groups. **E.** Comparison of the frequency of action potentials (APs) in NG, SMG-LA (−), and SMG-LA (+) groups. NG vs SMG-LA (−), *p* < 0.0001; SMG-LA (−) vs SMG-LA (+), *p* = 0.0021. **F.** Representative samples of mEPSCs in the neurons from NG, SMG-LA (−), and SMG-LA (+) groups. **G.** Comparison of the mEPSC frequency from NG, SMG-LA (−), and SMG-LA (+) groups. NG vs SMG-LA (−), *p* = 0.0022; SMG-LA (−) vs SMG-LA (+), *p* = 0.0472. **H.** Quantitative analyzes of the mEPSC amplitude from NG, SMG-LA (−), and SMG-LA (+) groups. Data are presented as mean±standard error of the mean (SEM). Two-way ANOVA or unpaired *t*-test was used for statistical comparison of differences, with values of *p* < 0.05 considered significant.

To further investigate how LA improved the rat brain function post SMG, whole-cell patch clamp assays were applied to assess the electrical activity of hippocampal CA1 pyramidal neurons (Figure 6A). The rest membrane potential collapsed in the SMG group compared with NG (Figure 6B), and the minimum current necessary to produce an action potential (rheobase) was increased (Figure 6C), indicating that the SMG model impaired the excitability of hippocampal CA1 pyramidal neurons. Less action potential (AP) could be induced with the identical injected current in SMG compared with NG treatment, especially from 60 pA to 140 pA, confirming that SMG suppressed the neuron excitability (Figure 6D-E). To note, oral administration of LA rescued SMG-induced excitability reduction of rat hippocampal neurons (Figure 6B-E). Next, the miniature excitatory postsynaptic currents (mEPSCs) were recorded and quantified. Intriguingly, it was the frequency of these events, but not the amplitude, that was remarkably restrained in the SMG group compared with NG (Figure 6F-H). The data suggested that under spontaneous conditions, the presynaptic signals were reduced in the SMG rats, which might be associated with the undue neuroinflammation and decreased synaptic protein expression (Figure 5). LA administration partially elevated the frequency of mEPSCs, but did not trigger amplitude change (Figure 6F-H), which was consistent with its protective role by promoting immune homeostasis and improving synaptic plasticity (Figure 5). All in all, rat hippocampal CA1 pyramidal neurons were affected by SMG with evidence of dysfunction as detected by whole-cell patch clamp assays, and the SMG-associated attenuated neuronal excitability could be partially restored by LA administration.

### LA suppresses inflammatory microglial activation by interacting with and inhibiting STAT1 phosphorylation at Tyr 701 and Ser 727

**Figure 7.**
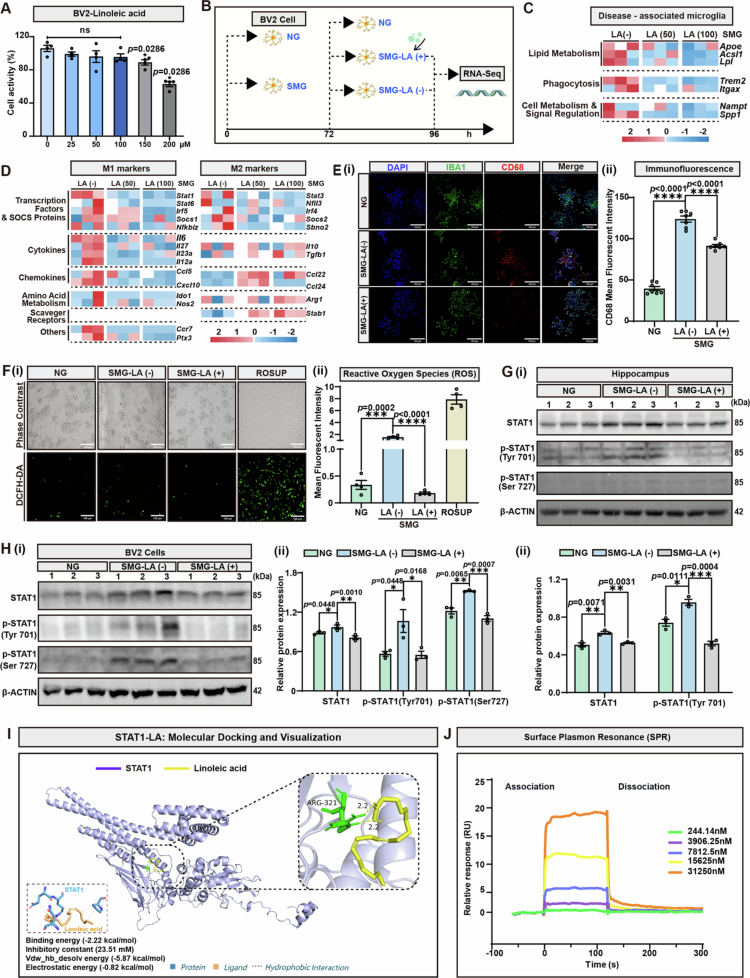
LA inhibits the activation of inflammatory microglia by binding to STAT1 and blocking its phosphorylation at Tyr 701 and Ser 727 sites. **A.** Schematic of the experimental design *in vitro*. BV2 microglial cells were cultured under SMG for 72 h, followed by the treatment with LA. After 24 h post-LA administration, cells were collected for RNA sequencing and other detection assays. **B.** Cell viability was determined by CCK8 assay with the exposure of LA at indicated concentrations for 24 h: 0 μM vs 150 μM, *p* = 0.0286; 0 μM vs 200 μM, *p* = 0.0286. **C.** RNA sequencing heatmap analysis of BV2 disease-associated microglia makers in SMG-LA (−), SMG-LA (50) (50 μM), or SMG-LA (100) (100 μM) group. **D.** RNA sequencing heatmap analysis of BV2 M1/M2 makers in SMG-LA (−), SMG-LA (50) (50 μM), or SMG-LA (100) (100 μM) group. **E.** (i) Immunofluorescent analysis for IBA1 and CD68 expression in BV2 (scale bars, 100 μm); (ii) Statistics on the mean fluorescence intensity of E(i). NG vs SMG-LA (−), *p* < 0.0001; SMG-LA (−) vs SMG-LA (50) (50 μM), *p* < 0.0001. **F.** Detection of reactive oxygen species (ROS) levels using DCFH-DA probe in BV2 cells. (i) Immunofluorescent analysis; (ii) Statistics on the mean fluorescence intensity of F(i). NG vs SMG-LA (−), *p* = 0.0002; SMG-LA (−) vs SMG-LA (50) (50 μM), *p* < 0.0001. Rosup as a positive control. **G.** (i) Immunoblot analysis of three independent individuals for t-STAT1, *p*-STAT1(Tyr 701) and *p*-STAT1(Ser 727) in the hippocampus. (ii) Relative protein expression of STAT1 (NG vs SMG-LA (−), *p* = 0.0071; SMG-LA (−) vs SMG-LA (50) (50 μM, *p* = 0.0031) and *p*-STAT1(Tyr 701) (NG vs SMG-LA (−), *p* = 0.0111; SMG-LA (−) vs SMG-LA (50) (50 μM, *p* = 0.0004) by grayscale analysis. **H.** (i) Immunoblot analysis of three independent samples for STAT1, *p*-STAT1(Tyr 701) and *p*-STAT1(Ser 727) in BV2 cells. (ii) Relative protein expression of STAT1 (NG vs SMG-LA (−), *p* = 0.0448; SMG-LA (−) vs SMG-LA (50) (50 μM, *p* = 0.001), *p*-STAT1(Tyr 701) (NG vs SMG-LA (−), *p* = 0.0448; SMG-LA (−) vs SMG-LA (50) (50 μM, *p* = 0.0168) and *p*-STAT1(Ser 727) (NG vs SMG-LA (−), *p* = 0.0065; SMG-LA (−) vs SMG-LA (50) (50 μM, *p* = 0.0007) by grayscale analysis. **I.** Molecular docking diagrams of LA binding to STAT1. **J.** Surface plasmon resonance (SPR) analysis of the interaction between LA and STAT1. Results are based on three independent biological replicates. Data are shown as the mean ± SD (rats’ sample) or mean ± SEM (BV2 sample). The analysis is performed using two-tailed unpaired Student’s *t* test. *p < 0.05, **p < 0.01, ***p < 0.001, ****p < 0.0001; ns no significance.

To elucidate how LA administration exerts neuroprotective effects, a 10⁻³ g microgravity environment comparable to that of the International Space Station for further *in vitro* experiments was produced with a special instrument (the gravity controller Gravite®) through two-axis rotation, the effectiveness of which has been confirmed previously.^37^ Given the metabolic incapacity of neurons to utilize lipids^38^ and the well-documented role of microglia as the exclusive immunoregulators governing both the initiation and resolution of neuroinflammation in the CNS,^39^ we postulated that LA might confer neuroprotection by fine-tuning microglial activation states. Cell viability screening based on the results of the CCK8 assays revealed that LA at concentrations ranging from 0 to 100 μM was suitable for functional studies of the microglial cell line BV2 (Figure 7A, Figure S8-ii). Based on previous studies,^40–42^ the optimal duration of SMG *in vitro* was set to four days to maintain the maximal cellular activity and responsiveness of BV2 cells, and LA was added on Day 3 post-SMG exposure (Figure 7B). To determine the dose-dependent effects of LA on microglial activation, BV2 cells were cultured with 50 μM (SMG-LA (50) group) or 100 μM (SMG-LA (100) group) LA under SMG conditions at Day 3, and RNA sequencing was performed on Day 4 post-SMG exposure to analyze the gene expression profiles. Transcriptomic analysis revealed that LA administration significantly downregulated the expression of disease-associated microglial (DAM) markers, including lipid metabolism genes (apolipoprotein E-*Apoe*, and acyl-CoA synthetase long-chain family member 1-*Acsl1*) and phagocytosis-related genes (triggering receptor expressed on myeloid cells 2-*Trem2*, and integrin alpha X-*Itgax*), as well as multiple proinflammatory indicators; in contrast, the expression of anti-inflammatory genes was strengthened by LA treatment (Figure 7C-D). These results suggested that LA remodeled the polarization pattern of microglia to alleviate neuroinflammation. KEGG pathway enrichment analysis revealed that compared with those in the SMG-LA (−) group, the genes differentially expressed in microglia in the 50 μM LA group were enriched in death- or survival-related pathways, including ferroptosis, AMPK signaling pathway, lipid metabolism and atherosclerosis, the PI3K-Akt signaling pathway, and PPAR signaling. Compared with treatment with SMG-LA (50), treatment with a higher dose of LA (the SMG-LA (100) group) altered the expression of genes involved in efferocytosis, cytokine-cytokine receptor interactions, the Ras signaling pathway, and the Hippo signaling pathway (Figure S9). Immunofluorescence assays also revealed that the enhanced expression of cluster of differentiation 68 (CD68) induced by SMG was markedly reversed by LA supplementation (Figure 7E), confirming that LA could block SMG-induced microglial activation. Concurrently, LA intervention significantly attenuated detrimental ROS production, as evidenced by the decreased ROS fluorescence intensity compared with that in the SMG-LA (−) group (Figure 7F), providing dual evidence of both the anti-inflammatory effects and the antioxidant effects of LA.

Signal transducer and activator of transcription 1 (STAT1) and its phosphorylated forms are key regulators of neuroinflammatory proinflammatory responses, mediating pathology by regulating proinflammatory cytokines, activating microglia/astrocytes, and amplifying inflammatory cascades to exacerbate neuronal injury,^43^^,^^44^ and the results of our KEGG analysis of our transcriptomic data also suggested the potential involvement of the STAT1 pathway in modulating microglial phenotype post-SMG treatment (Figure 7D). To determine whether LA inhibited neuroinflammation by regulating STAT1-mediated microglial activation, the protein levels of total STAT1 (t-STAT1) and phosphorylated STAT1 (*p*-STAT1) were measured. Immunoblot analysis revealed that LA administration significantly decreased the expression levels of both t-STAT1 and *p*-STAT1 (Tyr 701) in the hippocampus under SMG conditions (Figure 7G). Consistently, the supplement of LA also reversed the increased expression of t-STAT1, *p*-STAT1 (Tyr 701), and *p*-STAT1 (Ser 727) in SMG-treated BV2 cells (Figure 7H), demonstrating that LA suppressed the excessive activation of STAT1-associated microglia under *in vivo* or *in vitro* SMG conditions. Considering that metabolites act as inhibitors of certain transcription factors, we speculated that LA might interact with STAT1 and block its activation.

To further investigate if LA’s anti-inflammatory effect is STAT1-dependent, we treated SMG-induced microglia with LA, fludarabine (50 μM, a STAT1 inhibitor),^43^ and their combination, respectively (Figure S10A). qRT-PCR results showed that LA and fludarabine both reduced proinflammatory factors *Tnf-α*, *Ccl3*, and *Il-6*, but their combination did not further decrease this proinflammatory effect compared to LA alone (Figure S10B-i, ii, iii); both also increased anti-inflammatory *Arg1*, yet their combination did not further enhance this effect compared to LA alone (Figure S10B-iv). Additionally, LA’s ability to reduce ROS production was not enhanced when STAT1 was inhibited (Figure S10C-i, ii). Consistent with the central role of STAT1 in driving ISG expression, SMG-induced up-regulation of IFITM2, IFITM3, and MX1 was significantly inhibited by individual treatment with LA or fludarabine, while their combination showed no synergistic effect (Figure S10D).

Molecular docking analysis revealed the binding mode between LA and STAT1, demonstrating that a hydrogen bond (2.2 Å spacing) formed between LA and the arginine residue on STAT1 (Arg 321) (Figure 7I). Surface plasmon resonance (SPR) assays further characterized the interaction kinetics, yielding a dissociation equilibrium constant (KD) of 2.92 × 10⁻⁵ M, an association rate constant (ka) of 5.32 × 10³ M⁻¹s⁻¹, a dissociation rate constant (kd) of 1.55 × 10⁻¹ s⁻¹, and a maximum response (Rmax) of 20.46 RU with a chi-square value (χ²) of 0.208, confirming high-quality data fitting and reliable results (Figure 7J). The observed dose-dependent affinity of LA for STAT1 underscores the specificity and strength of their molecular interaction. Taken together, these results demonstrated that LA mitigated SMG-induced inflammatory microglial activation and oxidative damage by directly binding to STAT1 and inhibiting its phosphorylation.

## Discussion

Long-term exposure to microgravity induces malaise of motor perception and a lack of cognitive reserve in astronauts, which cannot be recovered in a short time after landing.^45^ Elucidating what causes decreases in postflight performance is important, as negative impacts on operator proficiency may lead to the failure of space shuttle tasks. Here, we confirm the role of gut dysbiosis in SMG-related encephalopathy through FMT and demonstrate that excessive consumption of LA by *Proteobacteria*, a protective metabolite to restrain neuroinflammation, induces cerebral dysfunction even after gravity returns to normal following short-term recovery. Oral administration of LA restricted STAT1-mediated microglial activation and rescued the synaptic plasticity impairments in the hippocampus, promoting the recovery of brain function during the gravitational transition from the SMG back to the NG (1 G).

The gut microbiome is a complex ecosystem that regulates various biological processes ranging from nutrient absorption to immune response and behavior alteration.^46–48^ Anaerobic bacteria dominate the colonic microbial population, the phylum of which is composed of *Proteobacteria* (mainly comprising *Enterobacteriaceae* and *Desulfovibrionaceae*), *Firmicutes* (mainly comprising *Lachnospiraceae* and *Ruminococcaceae*), *Bacteroidetes* (mainly comprising *Bacteroidaceae*, *Prevotellaceae,* and *Parabacteroides*), *Actinobacteria* (mainly comprising *Bifidobacteriaceae* and *Coriobacteriaceae*), and *Verrucomicrobia* (mainly comprising *Verrucomicrobiaceae*, *Opitutaceae,* and *Akkermansiaceae*), and the homeostasis of which serves as a holistic health indicator.^47^^,^^49^ A series of neuropsychiatric and neurodegenerative disorders are strongly associated with anomalous change of *Proteobacteria,* a type of inflammatory gut microbiota. An increased abundance of *Proteobacteria* with reduced fecal microbial diversity (especially with a reduction in *Firmicutes* abundance) was detected in patients with Alzheimer’s (AD)^50–52^ or Parkinson’s disease (PD),^53^ autism spectrum disorder (ASD),^54^ active major depressive disorder (A-MDD),^55^ and arteriosclerotic cerebral small vessel disease (ACSVD).^56^ These studies indicated that *Proteobacteria* might alter the gut microbial lipopolysaccharide (LPS) biosynthesis and phenylalanine-tyrosine metabolism, aggravating the cognitive impairments or social interaction deficits by activating TLR4/TNF-*α* signaling and promoting excessive neuroinflammation.

Additionally, based on different animal models and FMT experiments, a highly abundance of *Proteobacteria* might trigger cognitive deficits and depression-like behaviors. The overgrowth of *Proteobacteria* could be driven by chronic stress^57^ or sleep deprivation,^58^ long-term fiber deficiency,^59^ or a high-fat diet,^60^ and simultaneously, *Proteobacteria* could destroy intestinal barrier integrity, facilitate the translocation of detrimental metabolites from the gut mucosa to the serum, and ultimately result in microglial synaptic pruning dysfunction. Moreover, alterations in the abundance of *Proteobacteria* were detected in response to exposure to microgravity based on real-world studies or SMG models. A study at the International Space Station revealed that the *Parasutterella*, a member of the phylum *Proteobacteria*, increased in the gut microbiota and was associated with chronic intestinal inflammation in astronauts.^17^ Notably, compared with that in earth-bound individuals, the richness of the fecal microbiome in astronauts was consistently lower during and after spaceflight, which aligns with our findings in SMG-treated rats (Figure 2D–F). A head-down (−6°) tilt bed rest study of 60 days in humans revealed gut dysbiosis characterized by increased *Proteobacteria and Bacteroidetes*, which was linked to elevated antibiotic resistance in the microbiota.^61^ Hindlimb unloading for 8 weeks in rats extended the population of *E. coli*, a potentially pathogenic member of *Proteobacteria*, increasing intestinal mucosal permeability and the risk of colitis.^62^ In this study, we also revealed that the increased abundance of *Proteobacteria* in the rat gut could not return to normal within three days post-SMG treatment, which would hinder the recovery of brain function. These findings provide preliminary insights into the pathogenesis of microgravity-induced encephalopathy, but the messengers that mediate the crosstalk between the gut and brain remain unclear.

Emerging evidence suggests that metabolites derived from the intestinal microbiota, either beneficial or adverse for cerebral function, promote gut–brain communication by modulating the maturation and activation of microglia.^63^^,^^64^ Acetate, a favorable microbiome-derived short-chain fatty acid (SCFA), can restrain the neurodegenerative disease progression by maintaining mitochondrial integrity in microglia and increasing microglial phagocytosis, the process of which is deficient in germ-free mice with immature microglia.^65^ Butyrate, another advantageous SCFA primarily produced from fiber fermentation by the colonic microbiome, served as a histone deacetylase inhibitor that epigenetically suppressed the proinflammatory gene expression in microglia and improved memory ability in animal models.^66^ Tryptophan-related metabolites from gut commensal flora promoted the production of TGF-*α* and vascular endothelial growth Factor B (VEGF-*β*) from microglia, which could restrict the pathogenic activities of astrocytes via the ErbB1 receptor in an autoimmune encephalomyelitis (EAE) model.^67^ Intriguingly, tryptophan metabolism by the colonic microbiome might regulate behavior or motor disorders by indirectly affecting the production of proinflammatory kynurenine in the brain. Chronic restraint stress (CRS) is usually established to induce anxiety- and depression-like behaviors, and in this murine model, kynurenine levels in the CNS are negatively related to the abundance of *Enterorhabdus,* and *Parabacteroides* in the gut, which contribute to the pathogenesis of neuropsychiatric disorders by promoting inflammatory microglial activation via the aryl hydrocarbon receptor (AhR);^68^ moreover, the mechanism underlying these phenomena might involve the competitive consumption of tryptophan and the generation of other AhR ligands to restrain kynurenine production or the kynurenine-AhR axis in the host brain, while CRS-induced dysbiosis disrupts this protective signaling.^69^^,^^70^ In terms of SMG-induced encephalopathy, we found that the derivatives of LA in the gut, rather than foresaid SCFAs or tryptophan-related metabolites, decreased significantly post-SMG treatment (Figure 4), indicating their potential role in modulating cerebral function.

Although blood‒brain-barrier penetrable LA can be broken down through fatty acid *β*-oxidation, whose metabolites are likely products of the tricarboxylic acid (TCA) cycle, nearly 41% of LA in the brain is not used as an energy resource.^71^ It has been reported that LA or its derivatives can exert neuroprotective effects by improving neuronal function and remodeling microglial homeostasis. On the one hand, LA has neurotrophic and antioxidant properties and regulates mitochondrial quality control in neurons. LA and its metabolic derivatives, namely, arachidonic acid (ARA) and docosahexaenoic acid (DHA), serve as crucial structural components of neuronal phospholipids and help maintain synaptic membrane fluidity, which is indispensable for neurotransmitter release (e.g., glutamate and acetylcholine).^32^ LA, or safflower seed oil that contained more than 74% LA, increased the expression of brain-derived neurotrophic factor (BDNF) and suppressed oxidative injury, thus promoting neuronal survival in PD and other CNS diseases.^72–74^ The supplementation of LA, as well as its oxidized metabolites (OXLAMs) or its metabolic derivative ARA, was beneficial for neuronal morphogenesis by strengthening axonal outgrowth and synaptic plasticity, ameliorating depressive behavior, and improving cognitive function.^33^^,^^75^^,^^76^ LA also enhances the function of mitochondrial antioxidant and aerobic oxidation systems, such as by increasing the activity of oxidative phosphorylation enzymes (OXPHOS enzymes, including complex I and IV) and tTCA enzymes, including NADP + -dependent isocitrate dehydrogenase (ICDH) and alpha-ketoglutarate dehydrogenase (*α*-KGDH), thus promoting ATP production from mitochondria and improving oxidative stress and mitochondrial impairment in brain tissue.^77^ On the other hand, LA exerted critical immunoregulatory effects in the CNS by influencing microglial responses, the mechanisms of which were associated with the modulation of phagocytic ability and inflammatory pathways in microglia. The phagocytic capacity of mononuclear phagocytes can be enhanced by conjugated LA,^78^ which might help microglia clear tau aggregates or amyloid-*β* plaques in the CNS, thus potentially alleviating neurodegenerative pathologies. A recent study also revealed that LA suppressed excessive autophagic–lysosomal activation in microglia (but not sustained inhibition), which led to suitable clearance of myelin debris by microglia and ensured functional recovery from neural injury.^79^ In the case of LPS or saturated fatty acid stimulation, LA can competitively block TLR4 signaling or directly suppress NF-κB or ERK pathway-mediated inflammatory activation of microglia or other types of macrophages.^80–82^ Clinically, in patients with amyotrophic lateral sclerosis, plasma LA levels are negatively correlated with functional decline in the brain and positively correlated with survival rate.^83^

Notably, LA was found to be disadvantageous for inflammation progression in several studies. LA-derived diol 12, 13-DiHOME enhances NOD-like receptor thermal protein domain associated protein 3 (NLRP3) inflammasome activation in macrophages, exacerbating immunopathological injury.^84^ Furthermore, patients with dementia tended to have higher LA levels, which promoted the excessive production of ROS and played an adverse role in decreasing mitochondrial bioenergetics in AD.^85^ Consequently, LA might differentially regulate CNS function under distinct circumstances. Here, we demonstrated that LA administration was favorable for improving brain function post-SMG and revealed a new proinflammatory target (namely, STAT1) of LA in microglia. These data shed light on a unique mechanism through which LA derives SMG-activated microglia to a quiescent state and to exert neuroprotective effects. Additionally, we also reported that LA improved mucosal barrier function (Figure S7), potentially by inhibiting the translocation of detrimental metabolites from the gut to the brain. Notably, significantly increased STAT1 expression was detected in multiple immune cells (CD4, CD8, CD19, and PBMCs) in the peripheral blood of *NASA Twins Study* astronaut in- and post-flight,^6^ which is highly consistent with our observations in SMG-microglia.

This study elucidates the role of the gut-brain axis in microgravity-induced cerebral dysfunction. Notably, other biological mechanisms may exert independent or synergistic effects and thus merit consideration. Previous studies have demonstrated that both spaceflight and SMG can induce peripheral immune system dysfunction, characterized by increased total white blood cell counts and monocyte numbers. Concurrently, systemic inflammatory levels are elevated, with significant upregulation of proinflammatory cytokines such as IL-6 and TNF-α.^86^ Peripheral-derived inflammatory factors can penetrate the CNS through the impaired intestinal mucosal barrier or blood-brain barrier (BBB), further exacerbating neuroinflammation and thereby posing a potential threat to CNS function.^87^ This indicates that systemic inflammatory responses caused by peripheral immune disorders are involved in the regulation of cerebral function. In addition, spaceflight-associated stressors, including microgravity, space radiation, and hypoxic environments, can significantly promote the intracellular generation of ROS and reactive nitrogen species (RNS).^88^ Following short-term (5-13 days) spaceflight, the expression of antioxidant enzyme genes (e.g., superoxide dismutase 1 (SOD1) and glutathione peroxidase 1 (GPX1)) is markedly upregulated in astronauts' peripheral blood monocytes.^89^ Therefore, space stressors may also regulate cerebral function by inducing oxidative stress. This study has showed that linoleic acid (LA) exerts neuroprotective effects by inhibiting STAT1-mediated microglial activation. We hypothesize that LA may synergistically enhance these protective effects by improving peripheral immune cell function and boosting the body's antioxidant capacity. This potential mechanism awaits further verification in subsequent experiments.

Considering the animal welfare principles of the 3Rs (Replacement, Reduction, and Refinement), a priori power analysis was not performed for certain behavioral and electrophysiological experiments. Thia may influence the interpretation of statistical power for negative or borderline outcomes (e.g., trend-like changes in the Y-maze test). However, all omics data (including 16S rRNA sequencing and metabolomics) and key biochemical assays were performed with three independent biological replicates, thereby ensuring the reliability and reproducibility of the core dataset. In future related studies, the implementation of a priori power analysis for sample size estimation will help optimize experimental design and strengthen the robustness of the conclusions.

### Limitations

There are several limitations in the present study. First, while hindlimb unloading is currently recognized as a viable SMG model for research in cardiovascular, bone, brain, and intestinal fields, which still differs from the microgravity effects of the real space environment, future work should focus on developing better animal SMG models or sending more rodents into space. Second, we detected the alteration in the gut microbiome and metabolome, as well as the microglial response and synaptic plasticity, within three days post-SMG. However, all these dynamic changes during SMG exposure (such as those at 7, 14, and 21 days) should be measured to illustrate how LA regulates microglial activation and neuronal injury. Third, the influence of the gut microbiota or metabolites on mucosal immune responses should not be ignored. T cells (such as Th17 cells) can be activated in the gut mucosa and translocated to the CNS, which might also aggravate neuroinflammation. Finally, although LA has shown promise in preclinical studies, its safety profile, optimal dosage parameters, long-term efficacy in humans, and particularly its applicability to astronauts remain unclear. Future investigations should prioritize intervention protocols via dose-ranging studies and characterize LA-host metabolic interactions using humanized *in vitro* systems or clinical trials. Furthermore, it will be essential to systematically assess both acute and chronic outcomes, particularly in populations exposed to prolonged gravitational stress. These efforts will be essential to bridge the preclinical findings reported here with available countermeasures for maintaining gut–brain homeostasis in real-world applications.

## Conclusion

This study reveals that SMG promotes the proliferation of gut proinflammatory *Proteobacteria*, thus accelerating LA consumption and disrupting intestinal barrier integrity. This compromises LA-mediated inhibition of STAT1 phosphorylation in microglia, thereby driving their proinflammatory polarization and exacerbating neuroinflammation. SMG-induced consumption of LA may be closely associated with microgravity-associated cerebral dysfunction, including cognitive deficits and the emergence of anxiety-like behaviors. Dietary LA supplementation under the SMG could ameliorate brain function by rebuilding immune homeostasis in the hippocampus and mitigating synaptic impairment. These findings suggest that the “microbiota–metabolite–microglia” axis may represent a potential candidate target for protecting brain health under microgravity conditions, offering potential new research avenues for maintaining CNS health during prolonged exposure to microgravity. Notably, LA supplementation as a potential countermeasure for spaceflight applications necessitates further investigations encompassing dose optimization, comprehensive safety assessment and validation across multiple models to enable its translational development for clinical use.

## Materials and methods

### Cell, animal, and bacteria

The BV2 microglia cells (preserved in our laboratory) were cultured in Dulbecco's Modified Eagle Medium (DMEM, Servicebio) supplemented with 10% fetal bovine serum (FBS, Servicebio) and 1% penicillin-streptomycin (PS, Servicebio), maintained at 37 °C in a 5% CO₂ humidified incubator.

Male Sprague Dawley (SD) rats, weighing 150–200 g, were procured from the Experimental Animal Center of the Air Force Medical University. These animals were individually housed in standard cages and had ad libitum access to food and water.

*E. coli* (CICC 10389) and *P. aeruginosa* (CICC 21636) were purchased from the China Center for Industrial Culture Collection (CICC). The strains were cultured in Luria-Bertani (LB) broth (pH = 7.0) at 37 °C.

### Cell SMG model

A total of 1 × 10⁵ cells were seeded into a T25 cell culture flask, which was filled to maximum volume with culture medium to minimize hydrodynamic shear forces during rotational cultivation. The cells were cultivated using a 3D rotational bioreactor (Gravite® Controller, Space Bio-Laboratories Co., Ltd.), which operates by leveraging centrifugal forces generated through rotation to counteract gravitational forces acting on cells from the x, y, and z three-axis directions. Through actual measurement by a gravity acceleration sensor, these specific conditions generated a simulated environment with a gravitational acceleration of 10^−3^ G within 8 minutes. This mechanism effectively mimics the microgravity environment of space, creating a low-shear culture condition conducive to simulating physiological states in extraterrestrial environments.^90^^,^^91^

### Animal SMG model-hindlimb unloading (HU)

Using a −30° head-down hindlimb unloading model, in which the tail is secured with adhesive tape, rats bear a body load equivalent to 50% of their body weight with their forelimbs. This experimental configuration effectively mimics the physiological adaptations observed in the cardiovascular, skeletal, and muscular systems under microgravity conditions, and is widely acknowledged as the gold standard method for modeling space-induced weightlessness.^92^ In this setup, rats are housed individually in cages that permit unrestricted movement and ad libitum feeding, which significantly mitigates stress-related injuries associated with the SMG environment. To minimize the influence of confounding factors on brain function, we simultaneously set the horizontal tail suspension as the control group (NG group).

### Microglial depletion

Clodronate liposomes (10 μL, MCE, HY‐172202) were administered intracerebroventricularly (*i.c.v.*) every 24 h for 72 h.^93^^,^^94^ Control rats received an equal volume of artificial cerebrospinal fluid (ACSF). Depletion efficiency was verified by immunofluorescence, showing a significant decrease in Iba1⁺ cells in cortical brain sections.

### Animal behavioral experiments

Anxiety-like behaviors and cognitive functions in rats were evaluated using the open field test (OFT, 8 min), elevated plus maze (EPM, 5 min) test, and Y-maze test (5 min). Behavioral data were automatically analyzed using the Smart 3.0 Video Tracking System (Panlab SMART, Spain), with ethological parameters quantified via dedicated software.

### Electrophysiological Acquisition

In brief, rats were deeply anesthetized and transcardially perfused with 95% O_2_ and 5% CO_2_ oxygenated ice-cold NMDG cutting solution comprising (in mM): 93 NMDG, 93 HCl, 2.5 KCl, 1.2 NaH_2_PO_4_, 30 NaHCO_3_, 25 D-glucose, 20 HEPES, 5 Na-ascorbate, 2 thiourea, 3 Na-pyruvate, 10 MgSO_4_, and 0.5 CaCl_2_, pH 7.35 with NMDG or HCl. The brain was quickly removed and immersed in ice-cold NMDG cutting solution. Horizontal hippocampal slices of 300 μm thickness were cut with a Leica VT1200s vibratome (Leica Biosystems, Wetzlar, Germany), recovered at 34 °C for 10-13 min in NMDG cutting solution, and then maintained at 25 °C in oxygenated artificial cerebrospinal fluid (ACSF in mM: 126 NaCl, 2.5 KCl, 2 CaCl_2_,2 MgCl_2_, 26 NaHCO_3_, 1.25 NaH_2_PO_4_, and 10 glucose) for 1 h until electrophysiological recordings.

Slices were transferred to the recording chamber and superfused with oxygenated ACSF (~ 3 ml/min). Slices were visualized with infrared optics using an upright microscope equipped with a 40 × water-immersion lens (Olympus, BX51WI). Pyramidal cells from CA1 were identified by their location and morphology.

For whole-cell recording of action potentials (eAP), pipettes were filled with a solution containing (in mM): 135 K-gluconate, 5 KCl, 0.5 CaCl_2_, 10 HEPES, 2 Mg-ATP, 0.1 GTP, and 5 EGTA, 300 mOsm (pH adjusted to 7.3 with KOH). Current-evoked APs were elicited with 400-ms current injections at eight intensities (0, 20, 40, 60, 80, 100, 120, and 140 pA), with a 20-s trial interval. The frequency of AP was defined as numbers/time window.

For whole-cell recording of miniature excitatory postsynaptic currents (mEPSCs), pipettes were filled with a solution containing (in mM): 135 Cs-methanesulfonate, 10 KCl, 1 MgCl2, 0.2 EGTA, 4 Mg-ATP, 0.3 Na_2_GTP, 20 phosphocreatine, and 10 QX-314, (pH 7.3, at 285–290 mOsm). Cells were voltage clamped at -70 mV to monitor mEPSCs. Bicuculline (10 μM), DL-APV (50 μM) and TTX (1 μM) were used to block the GABAA, NMDA receptors, and Na^+^ channel, respectively. A 5-min recording duration was used for frequency and amplitude analyzes. Peak events were detected automatically using an amplitude threshold of 5 pA.

All signals were acquired with a MultiClamp 700B amplifier (Molecular Devices), filtered at 2 kHz, and sampled at 10 kHz with a Digidata 1440 A interface using Clampex 10.2 (Molecular Devices). Data were accepted when the series resistance fluctuated within 20% of initial values.

### qRT-PCR

Total RNA was extracted from cells/tissues using the Total RNA Maxi Kit (OMEGA, USA), and RNA concentration and purity were assessed to ensure they met optimal standards via spectrophotometric analysis (A260/A280 ratio: 1.8–2.0). Subsequently, cDNA was synthesized by reverse transcribing 1 μg of total RNA using the MightyScript First Strand cDNA Synthesis Master Mix (Sangon Biotech, China), following the manufacturer’s protocols. Quantitative real-time PCR (qPCR) was performed on the LightCycler® 480 System (Roche, Switzerland) using the SGExcel FastSYBR qPCR Kit (Sangon Biotech, China), with reaction conditions optimized for target genes and internal controls. Inflammatory cytokines & receptors PCR array plate (WC-MRNA0266-R, Wcgene® biotech) was used in this study. The primers used in this study are shown in Table 1.

**Table 1.** qRT-PCR primers used in this study.

Gene Name	Primer sequences (5’-3’)	Gene Name	Primer sequences (5’-3’)
rat-*Il-1β*-F	TTCTTTGAGGCTGACAGACC	mus-*Il-1β*-F	CCACTCACAGACAGCCACAA
rat-*Il-1β*-R	CGTCTTTCATCACACAGGAC	mus-*Il-1β*-R	GCTTGATGTGCTGCTGATGT
rat-*Tnf-α*-F	CCAGGTTCTCTTCAAGGGACAA	mus-*Tnf-α*-F	CAGGCGGTGCCTATGTCTC
rat-*Tnf-α*-R	CTCCTGGTATGAAATGGCAAATC	mus-*Tnf-α*-F	GCCATAGAACTGATGAGAGGGAG
rat-*Il-6*-F	ACTTCCAGCCAGTTGCCTTCTTG	mus-*Il-6*-F	TAGTCCTTCCTACCCCAATTTCC
rat-*Il-6*-R	GGTCTGTTGTGGGTGGTATCCTC	mus-*Il-6*-R	TTGGTCCTTAGCCACTCCTTC
rat-*Ccl3*-F	CATGGCGCTCTGGAACGAA	mus-*Ccl3*-F	GCTGCTTTGCCTACCTCTCC
rat-*Ccl3*-R	TGCCGTCCATAGGAGAAGCA	mus-*Ccl3*-R	GCTTGGCTGGTGAGTAGAGA
rat-*Il-10*-F	TGCCTTCAGCAGAGTGAAG	mus-*Il-10*-F	GCTCTTACTGACTGGCATGAG
rat-*Il-10*-R	TGCCTTCAGCAGAGTGAAG	mus-*Il-10*-R	CGCAGCTCTAGGAGCATGTG
rat-*Gapdh*-F	CCCTTCATTGACCTCAACTAC	mus-*Arg1*-F	AGCACTGAGGAAAGCTGGTC
rat-*Gapdh*-R	CTTCTCCA TGGTGGTGAAGAC	mus-*Arg1*-R	TACGTCTCGCAAGCCAATGT
		mus-*Gapdh*-F	CCCTTCATTGACCTCAACTAC
		mus-*Gapdh*-R	CTTCTCCA TGGTGGTGAAGAC

### Immunoblot

Total protein was extracted from cells/tissue samples using RIPA lysis buffer (supplemented with protease and phosphatase inhibitors, when necessary). Protein concentration was quantified using the BCA protein assay kit, following the manufacturer’s instructions. Equal amounts of protein samples were mixed with 5 × loading buffer, denatured by heating at 95–100°C for 5 min, and separated by SDS-PAGE using 1 × running buffer. Electrophoresis was performed at 80 V for the stacking gel and 100 V for the resolving gel, with a total run time of 2.5 hours. Proteins were then electrotransferred onto a PVDF membrane at a constant current of 300–400 mA for 1 hour. The membrane was blocked with 3% skim milk in TBST (0.1% Tween-20 in Tris-buffered saline) at room temperature for 1 hour to minimize non-specific binding. After washing, the membrane was incubated with primary antibodies (diluted in 5% BSA or skim milk in TBST, as recommended by the antibody supplier) overnight at 4 °C with gentle agitation. The next day, following thorough washing, the membrane was probed with horseradish peroxidase (HRP)-conjugated secondary antibodies (diluted in the same blocking buffer) for 1.5 hours at room temperature. Protein bands were visualized using an enhanced chemiluminescence (ECL) reagent, and images were captured using a gel documentation system. The antibodies used in this study are shown in Table 2.

**Table 2.** Immunoblot antibodies used in this study.

Antibodies name	Company	Cat no.
PSD95 Polyclonal antibody	Proteintech	30255-1-AP
DLG2 (PSD93) Polyclonal antibody	Proteintech	29457-1-AP
*α*-Synuclein Antibody	Cell Signaling	2642
STAT1 Polyclonal antibody	Proteintech	10144-2-AP
Phospho-STAT1 (Tyr701) Recombinant antibody	Proteintech	82674-10-RR
Phospho-STAT1 (Ser727) Polyclonal antibody	Proteintech	28977-1-AP
IFIT2 Polyclonal antibody	Proteintech	12604-1-AP
IFITM2 Polyclonal antibody	Proteintech	12769-1-AP
IFITM3 Polyclonal antibody	Proteintech	11714-1-AP
MX1 Polyclonal antibody	Proteintech	13750-1-AP
Recombinant Anti-beta Actin antibody (Rabbit mAb)	Servicebio	GB15003-100
HRP-conjugated AffiniPure Goat Anti-Rabbit IgG H&L	MCE	HY-P8001

The experimental procedures and cycling parameters are as follows:

**Table:** 

Step	Temperature	Time	Cycles
Pre-denaturation	95 °C	3 min	-
Denaturation	95 °C	5 s	35~40 cycles
Annealing/Extension	60 °C	20 s	35~40 cycles
Melting Curve Analysis	95 °C	15 s	-
Melting Curve Analysis	60 °C	1 min	-
Melting Curve Analysis	95 °C	15 s	-
Melting Curve Analysis	60 °C	15 s	-
Cooling	4℃	forever	-

### Immunofluorescence (IF)

Fixed cell or tissue samples were permeabilized with 0.05% Triton X-100 in phosphate-buffered saline (PBS) for 10 minutes at room temperature to enhance antibody accessibility. Samples were then blocked with 1% bovine serum albumin (BSA) in PBS for 1 hour at room temperature to minimize non-specific antibody binding. Target-specific primary antibodies were diluted in blocking buffer (1% BSA/PBS) and applied to the cells, which were incubated at 4 °C overnight with gentle orbital shaking to ensure uniform antibody binding. The next day, cells were washed three times with PBS to remove unbound primary antibodies. Species-matched fluorescent secondary antibodies (e.g., Alexa Fluor® conjugates, 1:500 dilution in blocking buffer) were added to the cells and incubated in the dark at room temperature for 1 hour to prevent photobleaching of the fluorophores. Nuclei were counterstained with 4',6-diamidino-2-phenylindole (DAPI, 1 μg/mL in PBS) for 30 minutes to visualize cellular nuclei. Following three additional PBS washes to remove excess DAPI, cells were mounted onto glass slides with an anti-fade mounting medium and sealed with coverslips. Fluorescence images were acquired using an Olympus confocal fluorescence microscope (FV3000). Exposure times and gain settings were optimized for each channel to avoid signal saturation, and images were captured using identical parameters across all samples to ensure consistency. The antibodies used in this study are shown in Table 3.

**Table 3.** Immunofluorescence antibodies used in this study.

Antibodies Name	Company	Cat No.
IBA1 Recombinant antibody	Proteintech	81728-1-RR
CD68/SR-D1 Antibody (FA-11)	Novus Biologicals	NBP2-33337
Recombinant Anti-ZO1 tight junction protein antibody	Servicebio	GB15195-100
Recombinant Anti-Occludin antibody	Servicebio	GB15149-100
Fluorescein (FITC)-conjugated Goat Anti-Rabbit IgG (H+L)	Proteintech	SA00003-2
Cy3-conjugated Goat Anti-Mouse IgG (H+L)	Proteintech	SA00009-1

### Hematoxylin-eosin (H&E) staining

Tissues from freshly euthanized rats were immediately harvested and fixed in 4% paraformaldehyde (PFA) at room temperature for 24–48 hours. Fixed tissues were dehydrated through graded ethanol (70%, 80%, 95%, 100%) (dehydrator, DIAPATH, Donatellos), cleared with xylene, and embedded in paraffin wax (embedding machine, Wuhan Junjie Electronics Co., Ltd, JB-P5). Using a microtome, tissue blocks were sectioned into 8-μm-thick slices, mounted onto glass slides, and air-dried overnight at 37 °C (microtome, Shanghai Leica Instruments Co., Ltd., RM2016). Before staining, sections were deparaffinized in xylene and rehydrated through a descending ethanol series (100%, 95%, 80%, 70%) to aqueous conditions. Sections were stained with hematoxylin for 5–8 minutes to color nuclei and basophilic structures blue-purple. After rinsing with tap water, they were differentiated in 1% hydrochloric acid in ethanol for 10–15 seconds and blued in Scott’s tap water substitute for 2–3 minutes. Following washing, cytoplasm and acidophilic structures were counterstained with eosin Y for 3–5 minutes, appearing red to pink. Stained sections were dehydrated in graded ethanol, cleared in xylene, and coverslipped with mounting medium. Images were acquired using a brightfield microscope (Olympus BX53) to visualize tissue morphology.

### Transmission electron microscopy (TEM)

Tissue samples collected were fixed with 2.5% glutaraldehyde and incubated at 4 °C overnight. After being rinsed with PBS, the samples underwent postfixation with 1% osmium tetroxide. Dehydration was carried out using a graded series of ethanol solutions. The samples were infiltrated with a resin and subjected to polymerization at the appropriate temperature. Ultrathin sections, 50–90 nm in thickness, were obtained from the polymerized resin blocks. Contrast enhancement of the sections was achieved through staining with uranyl and lead salts. The sections were mounted onto copper grids, excess liquid was removed, and they were air-dried. Subsequently, the sections were examined, and the acquired images were documented and analyzed on the electron microscopy stage.

### Fecal microbiota transplantation (FMT)

An antibiotic cocktail (Abx) was freshly prepared on the day of administration and incorporated into the rats' drinking water at final concentrations of 100 mg/L neomycin, 50 mg/L streptomycin, 100 mg/L penicillin, 50 mg/L vancomycin, and 100 mg/L metronidazole. This treatment regimen was applied to rats maintained on a standard diet for seven consecutive days.^95^

Fecal pellets were collected from rats 14 days post-hindlimb unloading and homogenized in phosphate-buffered saline (PBS) at a ratio of 1 g feces per 10 mL PBS. The homogenate was centrifuged at 500 rpm for 5 min at 4 °C, and the supernatant was collected for FMT.^96^ The prepared fecal suspension was administered via daily oral gavage at a volume of 500 μL for 14 consecutive days.

### High-throughput 16S rRNA gene sequencing of microbiota

As described in the previous figures, feces from rats in each group were collected at the corresponding time points (Figures 3‒4). Total genomic DNA was extracted from fecal samples using the TGuide S96 Magnetic Soil/Stool DNA Kit (Tiangen Biotech (Beijing) Co., Ltd.) according to the manufacturer’s instructions. The hypervariable region V3-V4 of the bacterial 16S rRNA gene was amplified with primer pairs 338F: 5'-ACTCCTACGGGAGGCAGCA-3' and 806 R: 5'-GGACTACHVGGGTWTCTAAT-3'. PCR products were checked on an agarose gel and purified using the Omega DNA purification kit (Omega Inc., Norcross, GA, USA). The purified PCR products were collected, and the paired ends (2 × 250 bp) were sequenced on the Illumina Novaseq 6000 platform.

### Bioinformatic analysis

**OTU cluster:** The bioinformatics analysis of this study was performed with the aid of the BMKCloud (http://www.biocloud.net/). According to the quality of single nucleotide, raw data were primarily filtered by Trimmomatic^97^ (version 0.33). Identification and removal of primer sequences was process by Cutadapt^98^ (version 1.9.1). PE reads obtained from previous steps were assembled by USEARCH^99^ (version 10) and followed by chimera removal using UCHIME (version 8.1). The high-quality reads generated from the above steps were used in the following analysis. Sequences with similarity > 97% were clustered into the same operational taxonomic unit (OTU) by USEARCH (version 10), and the OTUs containing less than 2 in all samples were filtered.

**ASV (dada2):** Clean reads were then conducted on feature classification to output ASVs (amplicon sequence variants) by dada2,^100^ and the ASVs with counts less than 2 in all samples were filtered. Taxonomy annotation of the OTUs was performed based on the Naive Bayes classifier in QIIME2^101^ using the SILVA database^102^ (release 138.1) with a confidence threshold of 70%. The *α*-diversity was calculated and displayed by the QIIME2 and R software, respectively. The *β*-diversity was determined to evaluate the degree of similarity of microbial communities from different samples using QIIME. Principal coordinate analysis (PCoA), heatmaps, UPGMA, and nonmetric multidimensional scaling (NMDS) were used to analyze the beta diversity. Furthermore, we employed Linear Discriminant Analysis (LDA) effect size (LEfSe)^103^ to test the significant taxonomic difference among groups. A logarithmic LDA score of 4.0 was set as the threshold for discriminative features. To explore the dissimilarities of the microbiome among different factors, a redundancy analysis (RDA) was performed in R using the package “vegan”. The ASV methods was used for the primary analysis presented in the figures.

### Gut microbiota metabolites sequencing

As described in the previous figure, feces from rats in each group were collected at the corresponding time points (Figure 4). The LC/MS system for gut microbiota metabolites sequencing metabolomics analysis consists of a Waters Acquity I-Class PLUS UPLC tandem with a Waters Xevo G2-XS QTof HRMS with a Waters Acquity UPLC HSS T3 (1.8 μm, 2.1 × 100 mm) column. Mobile phases are 0.1% formic acid in water (A) and 0.1% formic acid in acetonitrile (B) for both positive and negative ion modes. The Waters Xevo G2-XS QTOF HRMS, controlled by MassLynx V4.2, collects MS and MS/MS data in MSe mode, with dual-channel data acquisition at low (2 V) and high (10–40 V) collision energies in each cycle and a scanning frequency of 0.2 seconds. ESI ion source parameters include: capillary voltage 2000 V (+) or −1500 V (−), cone voltage 30 V, ion source temperature 150 °C, desolvent gas temperature 500 °C, backflush gas flow 50 L/h, and desolvation gas flow 800 L/h. Raw MassLynx V4.2 data are processed by Progenesis QI for peak extraction and alignment, and compounds are identified based on the online METLIN database and Biomarker's in-house library, with theoretical fragment identification and mass deviation within 100 ppm. After normalizing the original peak area by the total peak area, PCA and Spearman correlation analysis are used to assess sample repeatability and QC sample quality. Identified compounds are searched in KEGG, HMDB, and LipidMaps for classification and pathway information. Fold-change (FC), *T*-test *p*-values, and OPLS-DA VIP values are calculated, and differential metabolites are screened with criteria of FC > 1, *p* < 0.05, and VIP > 1. The significance of KEGG pathway enrichment for differential metabolites is calculated by a hypergeometric distribution test.

### Metabolite intervention model

*In vivo* model: Rats were housed under normal gravity conditions for 30 days. From Day 1 onward, they were continuously fed a diet supplemented with 10% (w/w) safflower seed oil (high linoleic acid content).^104^ On Day 31, hindlimb unloading was initiated to simulate microgravity, and this treatment was sustained until Day 58. The OFT, EPM test, and Y-maze test were conducted on Day 59, Day 60, and Day 61, respectively. After the behavioral tests, rats were euthanized, and samples, including colon and hippocampus, were collected. Adult Sprague-Dawley rats (200–250 g) were euthanized in accordance with AVMA guidelines (https://olaw.nih.gov/avma-guidelines-2020.htm) by graded exposure to compressed CO₂ (≥99% purity) at a displacement rate of 50% chamber volume per minute. Death was confirmed by a secondary physical method (cervical dislocation). The procedure was performed to minimize animal stress.

*In vitro* model: BV2 microglial cells were used as the research object. At 0 h, the cells were placed in a simulated microgravity device and continuously exposed to a simulated microgravity environment starting from 1 hour. At 73 h, the cells were treated with linoleic acid (Sigma, 60-33-3) at concentrations of 50 μM or 100 μM, respectively, and the treatment lasted until 96 hours. At 96 h, the levels of reactive oxygen species (ROS) and inflammatory phenotypes of the cells were detected; meanwhile, immunoblot assays were performed at the corresponding time points to analyze the expression of target proteins; at the end of the experiment, total RNA was extracted from the cells, and RNA-seq analysis was carried out to explore changes in the gene expression profile.

### Golgi staining

Rats’ brains were dissected post-deep anesthesia, washed with distilled water, and fixed in Servicebio's Golgi fixative (G1069-15ML/30ML) without perfusion. Single samples were stored in light-proof bottles at room temperature (RT). Tissues were stained with G1069-1 solution at 26℃ in the dark for 14 days, with dye changes every 3 days after the initial 48 h. Post-staining, tissues were treated with G1069-3 at RT for 1 h, then at 4℃ for 3 days with one solution change. 60 μm sections were cut via Leica VT1000S vibratome, mounted on G6012-1 slides, and developed with G1069-2 for 30 min. Slides were mounted with G1402 and scanned using 3DHISTECH Pannoramic scanners. Images were viewed via CaseViewer2.4.

### RNA sequencing

Total RNA was extracted from BV2 cells using a standard protocol, with purity (OD260/280, OD260/230) and integrity assessed by Nanodrop and Agilent 5400, respectively. For eukaryotic samples, poly (A) + mRNA was enriched using Oligo(dT) magnetic beads, fragmented by ionic interruption, and reverse-transcribed into first-strand cDNA using random hexamer primers. Second-strand cDNA synthesis, purification, end-repair, adenylation, and adapter ligation (containing P5/P7, index, and Rd1/Rd2 SP) were performed sequentially. Target cDNA fragments (250–300 bp) were size-selected by AMPure XP beads and amplified via PCR to construct the library. Library quality was validated using Qubit for quantification, Agilent 5400 for insert size analysis, and fluorescent quantitative PCR to ensure an effective concentration > 2 nM. Qualified libraries were pooled according to their concentrations and target data yield, followed by Illumina Novaseq PE150 sequencing using a synthesis-based approach, where fluorescently labeled dNTPs were used to generate sequence information by capturing emission signals during strand extension (https://www.biotree.cn).

### Intracellular reactive oxygen species (ROS) detection

BV2 microglial cells were seeded in T25 flasks, allowed to adhere, and cultured until a stable monolayer formed. After SMG treatment, metabolites and/or fludarabine (STAT1 inhibitor) were added, followed by an additional 24 h of incubation. Rosup (ROS inducer) served as the positive control. Post-incubation, the original medium was removed, and the cell monolayer was treated with the DCFH-DA probe diluted to an appropriate concentration in serum-free medium. Cells were further incubated in a cell incubator to facilitate probe entry. Subsequently, cells were gently washed with serum-free medium to remove unincorporated probe, then observed under a fluorescence microscope to measure intracellular fluorescence intensity. Higher fluorescence intensity indicates elevated intracellular ROS levels.

### Blood routine examination

Peripheral blood samples were collected from rats. Specifically, after the rats were appropriately anesthetized, approximately 0.5 mL of blood was drawn from the tail vein into tubes containing an anticoagulant (such as EDTA-K₂). The collected blood samples were then analyzed using a Sysmex XN-1000V hematology analyzer, in strict accordance with the manufacturer’s operating instructions. Before sample analysis, the instrument was calibrated with standard calibration materials provided by the manufacturer to ensure accurate and reliable results.

### Molecular docking and visualization

The 3D structure of LA (CAS: 60-33-3) was retrieved from PubChem (https://pubchem.ncbi.nlm.nih.gov) in SDF format. The ligand was subsequently energy-minimized using OpenBabel and converted to PDB format. The crystal structure of the receptor STAT1 (UniProt ID: P42225) was obtained from UniProt (https://www.uniprot.org). Molecular docking was performed using AutoDock,^105^ with the number of genetic algorithm (GA) runs set to 50. The conformation with the lowest binding energy was selected for further analysis. Protein-ligand interactions were visualized and analyzed using the Protein-Ligand Interaction Profiler (PLIP) and PyMOL.

### Surface plasmon resonance

The CM5 chip was activated with 0.4 M EDC and 0.1 M NHS at 10 μL/min for 7 minutes. STAT1 protein in 10 mM sodium acetate buffer (pH = 4.5) was immobilized on the activated chip via amine coupling at 10 μL/min until reaching 500–1000 resonance units. Unreacted sites were blocked with 1 M ethanolamine-HCl (pH = 8.5) at 10 μL/min for 7 minutes. LA solutions serially diluted in HBS-EP + buffer were injected into the STAT1-immobilized flow cell at 30 μL/min for 180 s for binding, followed by a 300-second dissociation phase. A reference flow cell without STAT1 was used for background correction. The binding curves were double-referenced. Affinity (KD) was calculated by fitting the curves to a 1:1 Langmuir model using Biacore T200 Evaluation Software. Experiments were done at 25 °C with three replicates.

### Statistical analysis

Results are based on three biological replicates. Data are shown as the mean ± SD (rats’ sample) or mean ± SEM (BV2 sample). Statistical analyzes were performed using GraphPad Prism 8 (San Diego, CA, USA). The analysis is performed using two-way ANOVA or two-tailed unpaired Student’s *t* test. *p < 0.05, **p < 0.01, ***p < 0.001, ****p < 0.0001; ns no significance.

## Supplementary Material

Supplementary figures.docx Supplementary figures.docx

## Data Availability

The datasets generated and/or analyzed during the current study are available in the NCBI repository, accessible via https://dataview.ncbi.nlm.nih.gov/object/PRJNA1310856?reviewer=1m44qjma4nu35jp38374apagdi. The data that support the findings of this study are available from the corresponding author-YL, upon reasonable request.

## Moral statement

This study was conducted in strict accordance with the recommendations of the Guidelines for the Care and Use of Laboratory Animals from Air Force Medical University. The protocol was approved by the Medical Ethics Committee of Air Force Medical University (protocol number: 202209054). All surgeries were performed under isoflurane anesthesia, and every effort was made to minimize animal pain.

## References

[1] Marfia G, Navone SE, Guarnaccia L, Campanella R, Locatelli M, Miozzo M, Perelli P, Della Morte G, Catamo L, Tondo P, et al. Space flight and central nervous system: friends or enemies? Challenges and opportunities for neuroscience and neuro-oncology. J Neurosci Res. 2022;100(9):1649–1663. doi: 10.1002/jnr.25066. 35678198 PMC9544848

[2] Sy MR, Keefe JA, Sutton JP, Wehrens XHT. Cardiac function, structural, and electrical remodeling by microgravity exposure. Am J Physiol Heart Circ Physiol. 2023;324(1):H1–H13. doi: 10.1152/ajpheart.00611.2022. 36399385 PMC9762974

[3] Grimm D, Grosse J, Wehland M, Mann V, Reseland JE, Sundaresan A, Corydon TJ. The impact of microgravity on bone in humans. Bone. 2016;87:44–56. doi: 10.1016/j.bone.2015.12.057. 27032715

[4] Van Ombergen A, Demertzi A, Tomilovskaya E, Jeurissen B, Sijbers J, Kozlovskaya IB, Parizel PM, Van de Heyning PH, Sunaert S, Laureys S, et al. The effect of spaceflight and microgravity on the human brain. J Neurol. 2017;264(Suppl 1):18–22. doi: 10.1007/s00415-017-8427-x. 28271409 PMC5610662

[5] Lv H, Yang H, Jiang C, Shi J, Chen RA, Huang Q, Shao D. Microgravity and immune cells. J R Soc Interface. 2023;20(199):20220869. doi: 10.1098/rsif.2022.0869. 36789512 PMC9929508

[6] Garrett-Bakelman FE, Darshi M, Green SJ, Gur RC, Lin L, Macias BR, McKenna MJ, Meydan C, Mishra T, Nasrini J, et al. The NASA twins study: a multidimensional analysis of a year-long human spaceflight. Science. 2019;364(6436):127–128. doi: 10.1126/science.aaw7086. . 30975860 PMC7580864

[7] Krittanawong C, Singh NK, Scheuring RA, Urquieta E, Bershad EM, Macaulay TR, Kaplin S, Dunn C, Kry SF, Russomano T, et al. Human health during space travel: state-of-the-art review. Cells. 2022;12(1):40. doi: 10.3390/cells12010040. 36611835 PMC9818606

[8] Hupfeld KE, McGregor HR, Reuter-Lorenz PA, Seidler RD. Microgravity effects on the human brain and behavior: dysfunction and adaptive plasticity. Neurosci Biobehav Rev. 2021;122:176–189. doi: 10.1016/j.neubiorev.2020.11.017. 33454290 PMC9650717

[9] Chen K, Li F, Zhang S, Chen Y, Ikezu TC, Li Z, Martens YA, Qiao W, Meneses A, Zhu Y, et al. Enhancing TREM2 expression activates microglia and modestly mitigates tau pathology and neurodegeneration. J Neuroinflammation. 2025;22(1):93. doi: 10.1186/s12974-025-03420-8. 40122810 PMC11931752

[10] Li Z, Wu J, Zhao T, Wei Y, Xu Y, Liu Z, Chen X. Microglial activation in spaceflight and microgravity: potential risk of cognitive dysfunction and poor neural health. Front Cell Neurosci. 2024;18:1296205. doi: 10.3389/fncel.2024.1296205. 38425432 PMC10902453

[11] Teng Y, Mu J, Xu F, Zhang X, Sriwastva MK, Liu QM, Li X, Lei C, Sundaram K, Hu X, et al. Gut bacterial isoamylamine promotes age-related cognitive dysfunction by promoting microglial cell death. Cell Host Microbe. 2022;30(7):944–960e948. doi: 10.1016/j.chom.2022.05.005. 35654045 PMC9283381

[12] Huang Y, Zhang X, Yu C, Liu Y, Kang H, Liu Y, Ni Y, Xia Y, Jiang Z, Chen J, et al. Lactobacillus acidophilus promotes cognitive function recovery via regulating microglial peroxisomal function in cerebral ischemia. Cell Host Microbe. 2025;33(9):1484–1501e1412. doi: 10.1016/j.chom.2025.07.018. 40812303

[13] Hamm PB, Nicogossian AE, Pool SL, Wear ML, Billica RD. Design and current status of the longitudinal study of astronaut health. Aviat Space Environ Med. 2000;71(6):564–570. 10870814

[14] Gonzalez E, Lee MD, Tierney BT, Lipieta N, Flores P, Mishra M, Beckett L, Finkelstein A, Mo A, Walton P, et al. Spaceflight alters host-gut microbiota interactions. NPJ Biofilms Microbiomes. 2024;10(1):71. doi: 10.1038/s41522-024-00545-1. 39209868 PMC11362537

[15] Ramos-Nascimento A, Grenga L, Haange SB, Himmelmann A, Arndt FS, Ly YT, Miotello G, Pible O, Jehmlich N, Engelmann B, et al. Human gut microbiome and metabolite dynamics under simulated microgravity. Gut Microbes. 2023;15(2):2259033. doi: 10.1080/19490976.2023.2259033. 37749878 PMC10524775

[16] Seidler RD, Mao XW, Tays GD, Wang T, Zu Eulenburg P. Effects of spaceflight on the brain. Lancet Neurol. 2024;23(8):826–835. doi: 10.1016/S1474-4422(24)00224-2. 38945144

[17] Turroni S, Magnani M, Kc P, Lesnik P, Vidal H, Heer M. Gut microbiome and space Travelers' health: state of the art and possible Pro/Prebiotic strategies for long-term space missions. Front Physiol. 2020;11:553929. doi: 10.3389/fphys.2020.553929. 33013480 PMC7505921

[18] Ibrahim Z, Khan NA, Siddiqui R, Qaisar R, Marzook H, Soares NC, Elmoselhi AB. Gut matters in microgravity: potential link of gut microbiota and its metabolites to cardiovascular and musculoskeletal well-being. Nutr Metab (Lond). 2024;21(1):66. doi: 10.1186/s12986-024-00836-6. 39123239 PMC11316329

[19] Xiong Y, Guo J, Yu W, Zeng D, Song C, Zhou L, Anatolyevna NL, Baranenko D, Xiao D, Lu W. Molecular mechanism of microgravity-induced intestinal flora dysbiosis on the abnormalities of liver and brain metabolism. Int J Mol Sci. 2025;26(7):3094. doi: 10.3390/ijms26073094. 40243802 PMC11988970

[20] Siddiqui R, Qaisar R, Khan NA, Alharbi AM, Alfahemi H, Elmoselhi A. Effect of microgravity on the gut microbiota bacterial composition in a hindlimb unloading model. Life (Basel). 2022;12(11):1865. doi: 10.3390/life12111865. 36431000 PMC9698145

[21] Voorhies AA, Mark Ott C, Mehta S, Pierson DL, Crucian BE, Feiveson A, Oubre CM, Torralba M, Moncera K, Zhang Y, et al. Study of the impact of long-duration space missions at the international space station on the astronaut microbiome. Sci Rep. 2019;9(1):9911. doi: 10.1038/s41598-019-46303-8. 31289321 PMC6616552

[22] Zhao Y, Ji G, Zhou S, Cai S, Li K, Zhang W, Yan N, Song B, Qu L. IGF2BP2-Shox2 axis regulates hippocampal-neuronal senescence to alleviate microgravity-induced recognition disturbance. iSci. 2024;27(6):109917. doi: 10.1016/j.isci.2024.109917. PMC11134919 38812544

[23] Masarapu Y, Cekanaviciute E, Andrusivova Z, Westholm JO, Bjorklund A, Fallegger R, Björklund Å, Badia-i-Mompel P, Boyko V, Vasisht S, et al. Spatially resolved multiomics on the neuronal effects induced by spaceflight in mice. Nat Commun. 2024;15(1):4778. doi: 10.1038/s41467-024-48916-8. 38862479 PMC11166911

[24] Yin YS, Zhu YB, Liu JL, Fan QC, Wu XR, Zhao S, Wang J, Li Y, Lu W. Long-term spaceflight composite stress induces depressive behaviors in model rats through disrupting hippocampus synaptic plasticity. CNS Neurosci Ther. 2024;30(3):e14438. doi: 10.1111/cns.14438. 37849237 PMC10916436

[25] Chen A, Teng C, Wei J, Wu X, Zhang H, Chen P, Cai D, Qian H, Zhu H, Zheng X. Gut microbial dysbiosis exacerbates long-term cognitive impairments by promoting intestinal dysfunction and neuroinflammation following neonatal hypoxia-ischemia. Gut Microbes. 2025;17(1):2471015. doi: 10.1080/19490976.2025.2471015. 40008452 PMC11866968

[26] Zhang L, Yin Z, Liu X, Jin G, Wang Y, He L, Li M, Pang X, Yan B, Jia Z, et al. Dietary emulsifier polysorbate 80 exposure accelerates age-related cognitive decline. Brain Behav Immun. 2024;119:171–187. doi: 10.1016/j.bbi.2024.03.052. 38565398

[27] Alvarez R, Stork CA, Sayoc-Becerra A, Marchelletta RR, Prisk GK, McCole DF. A simulated microgravity environment causes a sustained defect in epithelial barrier function. Sci Rep. 2019;9(1):17531. doi: 10.1038/s41598-019-53862-3. 31772208 PMC6879622

[28] Mercado-Perez A, Beyder A. Gut feelings: mechanosensing in the gastrointestinal tract. Nat Rev Gastroenterol Hepatol. 2022;19(5):283–296. doi: 10.1038/s41575-021-00561-y. 35022607 PMC9059832

[29] Vinken M. Hepatology in space: effects of spaceflight and simulated microgravity on the liver. Liver Int. 2022;42(12):2599–2606. doi: 10.1111/liv.15444. 36183343

[30] Smith MB, Chen H, Oliver BGG. The lungs in space: a review of current knowledge and methodologies. Cells. 2024;13(13):1154. doi: 10.3390/cells13131154. 38995005 PMC11240436

[31] Zihni C, Mills C, Matter K, Balda MS. Tight junctions: from simple barriers to multifunctional molecular gates. Nat Rev Mol Cell Biol. 2016;17(9):564–580. doi: 10.1038/nrm.2016.80. 27353478

[32] Tallima H, El Ridi R. Arachidonic acid: physiological roles and potential health benefits - A review. J Adv Res. 2018;11:33–41. doi: 10.1016/j.jare.2017.11.004. 30034874 PMC6052655

[33] da Costa Souza F, Grodzki ACG, Morgan RK, Zhang Z, Taha AY, Lein PJ. Oxidized linoleic acid metabolites regulate neuronal morphogenesis in vitro. Neurochem Int. 2023;164:105506. doi: 10.1016/j.neuint.2023.105506. 36758902 PMC10495953

[34] Bazinet RP, Laye S. Polyunsaturated fatty acids and their metabolites in brain function and disease. Nat Rev Neurosci. 2014;15(12):771–785. doi: 10.1038/nrn3820. 25387473

[35] Leikin-Frenkel A, Schnaider Beeri M, Cooper I. How alpha linolenic acid May sustain blood-brain barrier integrity and boost brain resilience against Alzheimer's disease. Nutrients. 2022;14(23):5091. doi: 10.3390/nu14235091. 36501121 PMC9737216

[36] Porcedda C, Manca C, Carta G, Piras F, Banni S, Sogos V, Murru E. Anti-neuroinflammatory effects of conjugated linoleic acid isomers, c9, t11 and t10, c12, on activated BV-2 microglial cells. Front Cell Neurosci. 2024;18:1442786. doi: 10.3389/fncel.2024.1442786. 39398647 PMC11466893

[37] Matsushita H, Isoguchi A, Okada M, Masuda T, Misumi Y, Ichiki Y, Ueda M, Ando Y. Amyloid fibril formation is suppressed in microgravity. Biochem Biophys Rep. 2021;25:100875. doi: 10.1016/j.bbrep.2020.100875. 33364446 PMC7750487

[38] Barber CN, Raben DM. Lipid metabolism crosstalk in the brain: glia and neurons. Front Cell Neurosci. 2019;13:212. doi: 10.3389/fncel.2019.00212. 31164804 PMC6536584

[39] Cowan M, Petri WA, Jr. Microglia: immune regulators of neurodevelopment. Front Immunol. 2018;9:2576. doi: 10.3389/fimmu.2018.02576. 30464763 PMC6234957

[40] Pala R, Cruciani S, Manca A, Garroni G, El Faqir MA, Lentini V, Capobianco G, Pantaleo A, Maioli M. Mesenchymal stem cell behavior under microgravity: from stress response to a premature senescence. Int J Mol Sci. 2023;24(9):7753. doi: 10.3390/ijms24097753. 37175460 PMC10178040

[41] Kim M, Jang G, Kim KS, Shin J. Detrimental effects of simulated microgravity on mast cell homeostasis and function. Front Immunol. 2022;13:1055531. doi: 10.3389/fimmu.2022.1055531. 36591304 PMC9800517

[42] Calcagno G, Jeandel J, Frippiat JP, Kaminski S. Simulated microgravity disrupts nuclear factor kappaB signaling and impairs murine dendritic cell phenotype and function. Int J Mol Sci. 2023;24(2):1720. doi: 10.3390/ijms24021720. 36675236 PMC9865583

[43] Zhao Y, Ma C, Chen C, Li S, Wang Y, Yang T, Stetler RA, Bennett MVL, Dixon CE, Shi Y. STAT1 contributes to Microglial/Macrophage inflammation and neurological dysfunction in a mouse model of traumatic brain injury. J Neurosci. 2022;42(39):7466–7481. doi: 10.1523/JNEUROSCI.0682-22.2022. 35985835 PMC9525171

[44] He H, Zhang X, He H, Xu G, Li L, Yang C, Liu Y, You Z. Microglial priming by IFN-gamma involves STAT1-mediated activation of the NLRP3 inflammasome. CNS Neurosci Ther. 2024;30(10):e70061. doi: 10.1111/cns.70061. 39392762 PMC11468839

[45] Moore ST, Dilda V, Morris TR, Yungher DA, MacDougall HG, Wood SJ. Long-duration spaceflight adversely affects post-landing operator proficiency. Sci Rep. 2019;9(1):2677. doi: 10.1038/s41598-019-39058-9. 30804413 PMC6389907

[46] Wu G, Xu T, Zhao N, Lam YY, Ding X, Wei D, Fan J, Shi Y, Li X, Ji S, et al. A core microbiome signature as an indicator of health. Cell. 2024;187(23):6550–6565e6511. doi: 10.1016/j.cell.2024.09.019. 39378879

[47] de Vos WM, Tilg H, Van Hul M, Cani PD. Gut microbiome and health: mechanistic insights. Gut. 2022;71(5):1020–1032. doi: 10.1136/gutjnl-2021-326789. 35105664 PMC8995832

[48] Wu C, Luo F, Zhu Y, Liu C, Chen Z, Wang X, Tan J. Deciphering the potential causal and prognostic relationships between gut microbiota and brain tumors: insights from genetics analysis and machine learning. Exploration (Beijing). 2025;5(4):e20240087. doi: 10.1002/EXP.20240087. 40873641 PMC12380072

[49] Cheng J, Williams JP, Zhou L, Wang PC, Sun LN, Li RH, An J. Ozone rectal insufflation mitigates chronic rapid eye movement sleep deprivation-induced cognitive impairment through inflammation alleviation and gut microbiota regulation in mice. Med Gas Res. 2024;14(4):213–224. doi: 10.4103/mgr.MEDGASRES-D-23-00036. 39073330 PMC11257187

[50] Liu P, Wu L, Peng G, Han Y, Tang R, Ge J, Zhang L, Jia L, Yue S, Zhou K, et al. Altered microbiomes distinguish Alzheimer's disease from amnestic mild cognitive impairment and health in a Chinese cohort. Brain Behav Immun. 2019;80:633–643. doi: 10.1016/j.bbi.2019.05.008. 31063846

[51] Hung CC, Chang CC, Huang CW, Nouchi R, Cheng CH. Gut microbiota in patients with Alzheimer's disease spectrum: a systematic review and meta-analysis. Aging. 2022;14(1):477–496. doi: 10.18632/aging.203826. 35027502 PMC8791218

[52] Zhao Q, Baranova A, Cao H, Zhang F. Gut microbiome and major depressive disorder: insights from two-sample mendelian randomization. BMC Psychiatry. 2024;24(1):493. doi: 10.1186/s12888-024-05942-6. 38977973 PMC11232322

[53] Sun MF, Zhu YL, Zhou ZL, Jia XB, Xu YD, Yang Q, Cui C, Shen Y. Neuroprotective effects of fecal microbiota transplantation on MPTP-induced Parkinson's disease mice: gut microbiota, glial reaction and TLR4/TNF-alpha signaling pathway. Brain Behav Immun. 2018;70:48–60. doi: 10.1016/j.bbi.2018.02.005. 29471030

[54] Korteniemi J, Karlsson L, Aatsinki A. Systematic review: autism spectrum disorder and the gut microbiota. Focus (Am Psychiatr Publ). 2024;22(2):242–251. doi: 10.1176/appi.focus.24022008. 38680985 PMC11046714

[55] Jiang H, Ling Z, Zhang Y, Mao H, Ma Z, Yin Y, Wang W, Tang W, Tan Z, Shi J, et al. Altered fecal microbiota composition in patients with major depressive disorder. Brain Behav Immun. 2015;48:186–194. doi: 10.1016/j.bbi.2015.03.016. 25882912

[56] Huang J, Liu S, Li P, Wei L, Lin G, Lin J, Luo Y, Mao Y, Ruan H, Qin B, et al. Multi-omics analysis of gut-brain axis reveals novel microbial and neurotransmitter signatures in patients with arteriosclerotic cerebral small vessel disease. Pharmacol Res. 2024;208:107385. doi: 10.1016/j.phrs.2024.107385. 39245190

[57] Hao W, Ma Q, Wang L, Yuan N, Gan H, He L, Li X, Huang J, Chen J. Gut dysbiosis induces the development of depression-like behavior through abnormal synapse pruning in microglia-mediated by complement C3. Microbiome. 2024;12(1):34. doi: 10.1186/s40168-024-01756-6. 38378622 PMC10877840

[58] Yin C, Zhang M, Jin S, Zhou Y, Ding L, Lv Q, Huang Z, Chen J, Wang P, You Q. Mechanism of salvia miltiorrhiza bunge extract to alleviate chronic sleep deprivation-induced cognitive dysfunction in rats. Phytomedicine. 2024;130:155725. doi: 10.1016/j.phymed.2024.155725. 38772181

[59] Shi H, Ge X, Ma X, Zheng M, Cui X, Pan W, Yang X, Zhang P, Hu M, Tang R, et al. A fiber-deprived diet causes cognitive impairment and hippocampal microglia-mediated synaptic loss through the gut microbiota and metabolites. Microbiome. 2021;9(1):223. doi: 10.1186/s40168-021-01172-0. 34758889 PMC8582174

[60] Wang M, Sun P, Chai X, Liu YX, Li L, Zheng W, Chen S, Zhu X, Zhao S. Reconstituting gut microbiota-colonocyte interactions reverses diet-induced cognitive deficits: the beneficial of eucommiae cortex polysaccharides. Theranostics. 2024;14(12):4622–4642. doi: 10.7150/thno.99468. 39239516 PMC11373620

[61] Li Y, Liu Z, Luo G, Lan H, Chen P, Du R, Jing G, Cui X, Han Y, Xu J, et al. Effects of 60 days of 6 degrees head-down bed rest on the composition and function of the human gut microbiota. iSci. 2023;26(5):106615. doi: 10.1016/j.isci.2023.106615. PMC10214410 37250329

[62] Yang Y, Qu C, Liang S, Wang G, Han H, Chen N, Wang X, Luo Z, Zhong C, Chen Y, et al. Estrogen inhibits the overgrowth of escherichia coli in the rat intestine under simulated microgravity. Mol Med Rep. 2018;17(2):2313–2320. 29207065 10.3892/mmr.2017.8109 PMC5783461

[63] Dalile B, Van Oudenhove L, Vervliet B, Verbeke K. The role of short-chain fatty acids in microbiota-gut-brain communication. Nat Rev Gastroenterol Hepatol. 2019;16(8):461–478. doi: 10.1038/s41575-019-0157-3. 31123355

[64] Liu L, Wang H, Chen X, Zhang Y, Zhang H, Xie P. Gut microbiota and its metabolites in depression: from pathogenesis to treatment. EBioMedicine. 2023;90:104527. doi: 10.1016/j.ebiom.2023.104527. 36963238 PMC10051028

[65] Erny D, Dokalis N, Mezo C, Castoldi A, Mossad O, Staszewski O, Mezö C, Frosch M, Villa M, Fuchs V, et al. Microbiota-derived acetate enables the metabolic fitness of the brain innate immune system during health and disease. Cell Metab. 2021;33(11):2260–2276e2267. doi: 10.1016/j.cmet.2021.10.010. 34731656

[66] Kopczynska J, Kowalczyk M. The potential of short-chain fatty acid epigenetic regulation in chronic low-grade inflammation and obesity. Front Immunol. 2024;15:1380476. doi: 10.3389/fimmu.2024.1380476. 38605957 PMC11008232

[67] Rothhammer V, Borucki DM, Tjon EC, Takenaka MC, Chao CC, Ardura-Fabregat A, de Lima KA, Gutiérrez-Vázquez C, Hewson P, Staszewski O, et al. Microglial control of astrocytes in response to microbial metabolites. Nature. 2018;557(7707):724–728. doi: 10.1038/s41586-018-0119-x. 29769726 PMC6422159

[68] Deng Y, Zhou M, Wang J, Yao J, Yu J, Liu W, Wu L, Gao R. Involvement of the microbiota-gut-brain axis in chronic restraint stress: disturbances of the kynurenine metabolic pathway in both the gut and brain. Gut Microbes. 2021;13(1):1–16. doi: 10.1080/19490976.2020.1869501. PMC7872056 33535879

[69] Peesh P, Blasco-Conesa MP, El Hamamy A, Khan R, Guzman GU, Honarpisheh P, Mohan EC, Goodman GW, Nguyen JN, Banerjee A, et al. Benefits of equilibrium between microbiota- and host-derived ligands of the aryl hydrocarbon receptor after stroke in aged Male mice. Nat Commun. 2025;16(1):1767. doi: 10.1038/s41467-025-57014-2. 39971928 PMC11839985

[70] Garrison AM, Parrott JM, Tunon A, Delgado J, Redus L, O'Connor JC. Kynurenine pathway metabolic balance influences microglia activity: targeting kynurenine monooxygenase to dampen neuroinflammation. Psychoneuroendocrinology. 2018;94:1–10. doi: 10.1016/j.psyneuen.2018.04.019. 29734055 PMC5995655

[71] DeMar JC, Jr, Lee HJ, Ma K, Chang L, Bell JM, Rapoport SI, Bazinet RP. Brain elongation of linoleic acid is a negligible source of the arachidonate in brain phospholipids of adult rats. Biochim Biophys Acta. 2006;1761(9):1050–1059. doi: 10.1016/j.bbalip.2006.06.006. 16920015

[72] Barmak MJ, Nouri E, Shahraki MH, Ghalamfarsa G, Zibara K, Delaviz H, Ghanbari A. Safflower seed oil, a rich source of linoleic acid, stimulates hypothalamic neurogenesis in vivo. Anat Cell Biol. 2023;56(2):219–227. doi: 10.5115/acb.22.220. 36967238 PMC10319475

[73] Tang KS. Protective effect of arachidonic acid and linoleic acid on 1-methyl-4-phenylpyridinium-induced toxicity in PC12 cells. Lipids Health Dis. 2014;13:197. doi: 10.1186/1476-511X-13-197. 25522984 PMC4320435

[74] Mbiydzenyuy NE, Ninsiima HI, Valladares MB, Pieme CA. Zinc and linoleic acid pre-treatment attenuates biochemical and histological changes in the midbrain of rats with rotenone-induced parkinsonism. BMC Neurosci. 2018;19(1):29. doi: 10.1186/s12868-018-0429-9. 29739324 PMC5941606

[75] Hennebelle M, Morgan RK, Sethi S, Zhang Z, Chen H, Grodzki AC, Lein PJ, Taha AY. Linoleic acid-derived metabolites constitute the majority of oxylipins in the rat pup brain and stimulate axonal growth in primary rat cortical neuron-glia co-cultures in a sex-dependent manner. J Neurochem. 2020;152(2):195–207. doi: 10.1111/jnc.14818. 31283837 PMC6949423

[76] Yu H, Song Y, Lou M, Shen S. Mitigation and mechanism of low dose linoleic acid on depression caused by disorder of gut microbiome. Nutr Neurosci. 2025;28(2):245–262. doi: 10.1080/1028415X.2024.2366648. 38963806

[77] Aydin B, Guler Sahin C, Sekeroglu V, Atli Sekeroglu Z. Conjugated linoleic acid protects brain mitochondrial function in acrolein induced Male rats. Toxicol Mech Methods. 2021;31(9):674–679. doi: 10.1080/15376516.2021.1952673. 34238125

[78] Cho MH, Kang JH, Yang MP. Immunoenhancing effect of trans-10, cis-12 conjugated linoleic acid on the phagocytic capacity and oxidative burst activity of canine peripheral blood phagocytes. Res Vet Sci. 2008;85(2):269–278. doi: 10.1016/j.rvsc.2007.12.005. 18234254

[79] Zhou LQ, Dong MH, Hu ZW, Tang Y, Chu YH, Chen M, Yang S, Wu L, Wang W, Qin C, et al. Staged suppression of microglial autophagy facilitates regeneration in CNS demyelination by enhancing the production of linoleic acid. Proc Natl Acad Sci U S A. 2023;120(1):e2209990120. doi: 10.1073/pnas.2209990120. 36577069 PMC9910603

[80] Lowry JR, Marshall N, Wenzel TJ, Murray TE, Klegeris A. The dietary fatty acids alpha-linolenic acid (ALA) and linoleic acid (LA) selectively inhibit microglial nitric oxide production. Mol Cell Neurosci. 2020;109:103569. doi: 10.1016/j.mcn.2020.103569. 33161065

[81] Tu TH, Kim H, Yang S, Kim JK, Kim JG. Linoleic acid rescues microglia inflammation triggered by saturated fatty acid. Biochem Biophys Res Commun. 2019;513(1):201–206. doi: 10.1016/j.bbrc.2019.03.047. 30952426

[82] Ikeguchi S, Izumi Y, Kitamura N, Kishino S, Ogawa J, Akaike A, Kume T. Inhibitory effect of the gut microbial linoleic acid metabolites, 10-oxo-trans-11-octadecenoic acid and 10-hydroxy-cis-12-octadecenoic acid, on BV-2 microglial cell activation. J Pharmacol Sci. 2018;138(1):9–15. doi: 10.1016/j.jphs.2018.06.015. 30243517

[83] Bjornevik K, Cortese M, Furtado JD, Paganoni S, Schwarzschild MA, Cudkowicz ME, Ascherio A. Association of polyunsaturated fatty acids and clinical progression in patients with ALS: post hoc analysis of the EMPOWER trial. Neurology. 2023;101(7):e690–e698. doi: 10.1212/WNL.0000000000207485. 37344230 PMC10437021

[84] Valencia R, Kranrod JW, Fang L, Soliman AM, Azer B, Clemente-Casares X, Clemente‐Casares X, Seubert JM. Linoleic acid-derived diol 12, 13-DiHOME enhances NLRP3 inflammasome activation in macrophages. FASEB J. 2024;38(13):e23748. doi: 10.1096/fj.202301640RR.38940767

[85] Amick KA, Mahapatra G, Gao Z, Dewitt A, Craft S, Jain M, Molina AJA. Plasma glycocholic acid and linoleic acid identified as potential mediators of mitochondrial bioenergetics in Alzheimer's dementia. Front Aging Neurosci. 2022;14:954090. doi: 10.3389/fnagi.2022.954090. 36212044 PMC9540364

[86] Kim J, Tierney BT, Overbey EG, Dantas E, Fuentealba M, Park J, Narayanan SA, Wu F, Najjar D, Chin CR, et al. Single-cell multi-ome and immune profiles of the Inspiration4 crew reveal conserved, cell-type, and sex-specific responses to spaceflight. Nat Commun. 2024;15(1):4954. doi: 10.1038/s41467-024-49211-2. 38862516 PMC11166952

[87] Wang C, Fan X, Shi Y, Tang F. Radiation-induced brain injury with special reference to astrocytes as a therapeutic target. J Integr Neurosci. 2025;24(3):25907. doi: 10.31083/JIN25907. 40152565

[88] Mao XW, Pecaut MJ, Stanbouly S, Nelson G. Oxidative stress, neuroinflammation, and the blood-brain barrier biomarkers on the brain response to spaceflight. Life Sci Space Res (Amst). 2024;43:22–28. doi: 10.1016/j.lssr.2024.08.001. 39521489

[89] Correction to: cell-free mitochondrial DNA as a potential biomarker for Astronauts' health. J Am Heart Assoc. 2021;10(24):e020771. doi: 10.1161/JAHA.121.020771. 34666498 PMC8751818

[90] Mitsuhara T, Takeda M, Yamaguchi S, Manabe T, Matsumoto M, Kawahara Y, Yuge L, Kurisu K. Simulated microgravity facilitates cell migration and neuroprotection after bone marrow stromal cell transplantation in spinal cord injury. Stem Cell Res Ther. 2013;4(2):35. doi: 10.1186/scrt184. 23548163 PMC3706926

[91] Kawahara Y, Manabe T, Matsumoto M, Kajiume T, Matsumoto M, Yuge L. LIF-free embryonic stem cell culture in simulated microgravity. PLoS One. 2009;4(7):e6343. doi: 10.1371/journal.pone.0006343. 19626124 PMC2710515

[92] Globus RK, Morey-Holton E. Hindlimb unloading: rodent analog for microgravity. J Appl Physiol (1985). 2016;120(10):1196–1206. doi: 10.1152/japplphysiol.00997.2015. 26869711

[93] Hanlon LA, Raghupathi R, Huh JW. Depletion of microglia immediately following traumatic brain injury in the pediatric rat: implications for cellular and behavioral pathology. Exp Neurol. 2019;316:39–51. doi: 10.1016/j.expneurol.2019.04.004. 30980832 PMC6544393

[94] Han X, Li Q, Lan X, El-Mufti L, Ren H, Wang J. Microglial depletion with clodronate liposomes increases proinflammatory cytokine levels, induces astrocyte activation, and damages blood vessel integrity. Mol Neurobiol. 2019;56(9):6184–6196. doi: 10.1007/s12035-019-1502-9. 30734229 PMC6684378

[95] Li D, Feng Y, Tian M, Ji J, Hu X, Chen F. Gut microbiota-derived inosine from dietary barley leaf supplementation attenuates colitis through PPARgamma signaling activation. Microbiome. 2021;9(1):83. doi: 10.1186/s40168-021-01028-7. 33820558 PMC8022418

[96] Wang Y, Tang J, Lv Q, Tan Y, Dong X, Liu H, Zhao N, He Z, Kou Y, Dai L. Establishment and resilience of transplanted gut microbiota in aged mice. iSci. 2022;25(1):103654. doi: 10.1016/j.isci.2021.103654. PMC8733228 35024588

[97] Bolger AM, Lohse M, Usadel B. Trimmomatic: a flexible trimmer for illumina sequence data. Bioinformatics. 2014;30(15):2114–2120. doi: 10.1093/bioinformatics/btu170. 24695404 PMC4103590

[98] Kechin A, Boyarskikh U, Kel A, Filipenko M. cutPrimers: a new tool for accurate cutting of primers from reads of targeted next generation sequencing. J Comput Biol. 2017;24(11):1138–1143. doi: 10.1089/cmb.2017.0096. 28715235

[99] Edgar RC. UPARSE: highly accurate OTU sequences from microbial amplicon reads. Nat Methods. 2013;10(10):996–998. doi: 10.1038/nmeth.2604. 23955772

[100] Callahan BJ, McMurdie PJ, Rosen MJ, Han AW, Johnson AJ, Holmes SP. DADA2: high-resolution sample inference from illumina amplicon data. Nat Methods. 2016;13(7):581–583. doi: 10.1038/nmeth.3869. 27214047 PMC4927377

[101] Bolyen E, Rideout JR, Dillon MR, Bokulich NA, Abnet CC, Al-Ghalith GA, Alexander H, Alm EJ, Arumugam M, Asnicar F, et al. Reproducible, interactive, scalable and extensible microbiome data science using QIIME 2. Nat Biotechnol. 2019;37(8):852–857. doi: 10.1038/s41587-019-0209-9. 31341288 PMC7015180

[102] Quast C, Pruesse E, Yilmaz P, Gerken J, Schweer T, Yarza P, Peplies J, Glöckner FO. The SILVA ribosomal RNA gene database project: improved data processing and web-based tools. Nucleic Acids Res. 2013;41(Database issue):D590–596. doi: 10.1093/nar/gks1219. 23193283 PMC3531112

[103] Segata N, Izard J, Waldron L, Gevers D, Miropolsky L, Garrett WS, Huttenhower C. Metagenomic biomarker discovery and explanation. Genome Biol. 2011;12(6):R60. doi: 10.1186/gb-2011-12-6-r60. 21702898 PMC3218848

[104] Zhu S, Pang J, Nguyen A, Tan C, Tso A, Huynh T, Gu Y, Gustafsson AB, Vaz FM, Evans SM, et al. Temporal effects of safflower oil diet-based linoleic acid supplementation on barth syndrome cardiomyopathy. Circulation. 2024;149(10):790–793. doi: 10.1161/CIRCULATIONAHA.123.065414. 38437482 PMC10914323

[105] Forli S, Huey R, Pique ME, Sanner MF, Goodsell DS, Olson AJ. Computational protein-ligand docking and virtual drug screening with the AutoDock suite. Nat Protoc. 2016;11(5):905–919. doi: 10.1038/nprot.2016.051. 27077332 PMC4868550

