# Supplementary material for: Simulated microgravity induces cerebral dysfunction by disturbing protective microbiota-metabolite-microglia signaling across the gut‒brain axis
Source: Gut Microbes. 2026 Feb 23;18(1):2635820. doi: 10.1080/19490976.2026.2635820 (PMC12931916; doi:10.1080/19490976.2026.2635820)
Supplement: Supplementary figures.docx [file KGMI_A_2635820_SM3189.docx]

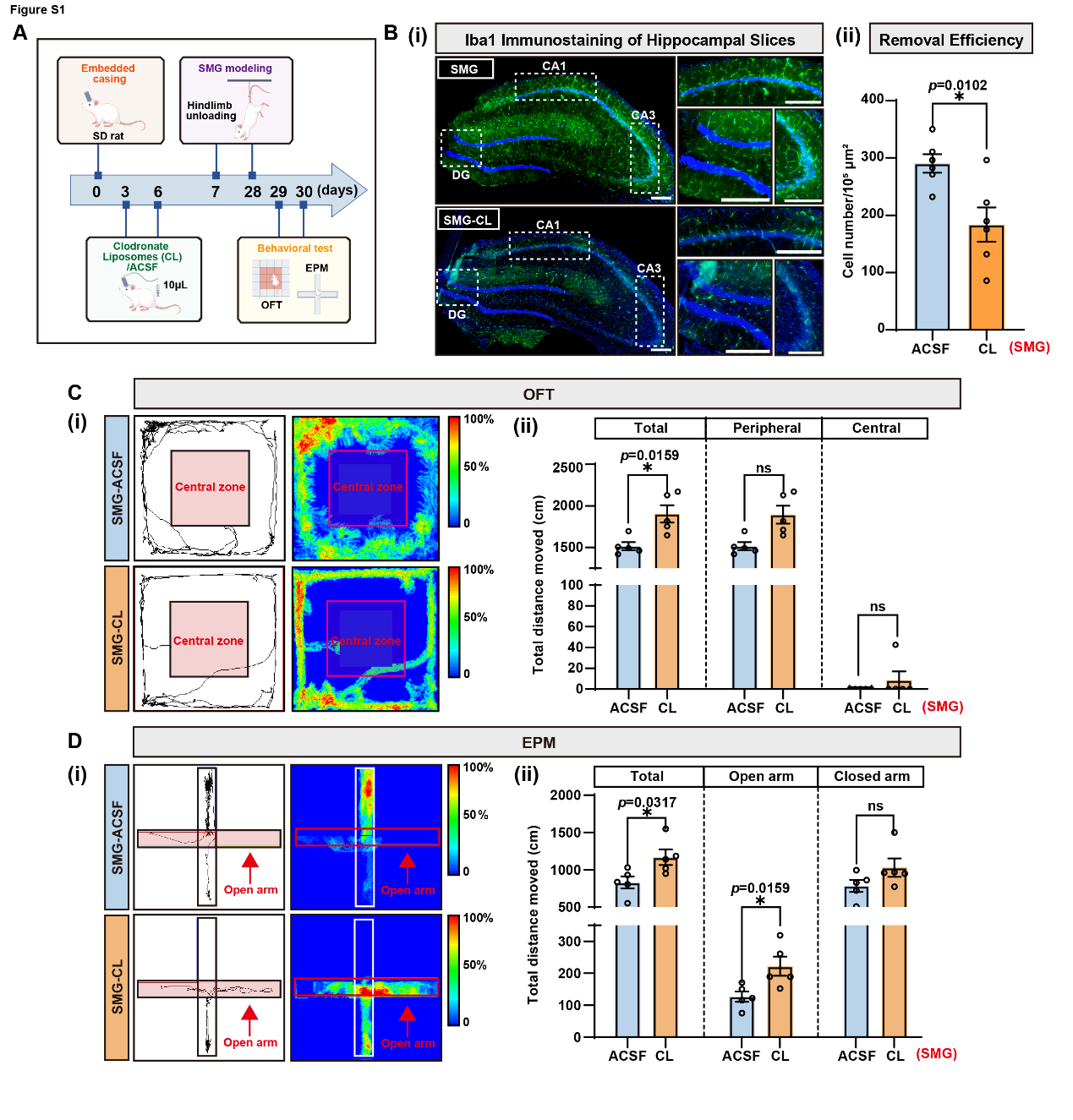


**Figure S1. Microglial depletion ameliorates SMG-induced brain dysfunction.**

**A.** Flowchart of microglial depletion in the brain using clodronate liposomes (CL). After intracranial cannulation in rats, injections of CL or artificial cerebrospinal fluid (ACSF) were administered on days 3 to 6, respectively. Hindlimb unloading was performed from day 7 to day 28, and OFT and EPM behavioral tests were conducted on days 29 and 30.

**B.** **(i)** Iba1^+^ microglia in hippocampal slices from SMG-treated rats receiving ACSF or CL (scale bar, 100 μm); (ii) Microglial density quantification in different groups.

**C.** OFT was performed on day 29 (n = 5). (i) Movement trajectories of rats in OFT; (ii) Distance moved in the total path (*p* = 0.0159), peripheral zone (ns), and central zone in OFT (ns).

**D.** EPM test was performed on day 30 (n = 5). (i) Movement trajectories of rats in EPM; (ii) Distance moved in total path (*p* = 0.0317), open arm (*p* = 0.0159), and closed arm (ns).

**
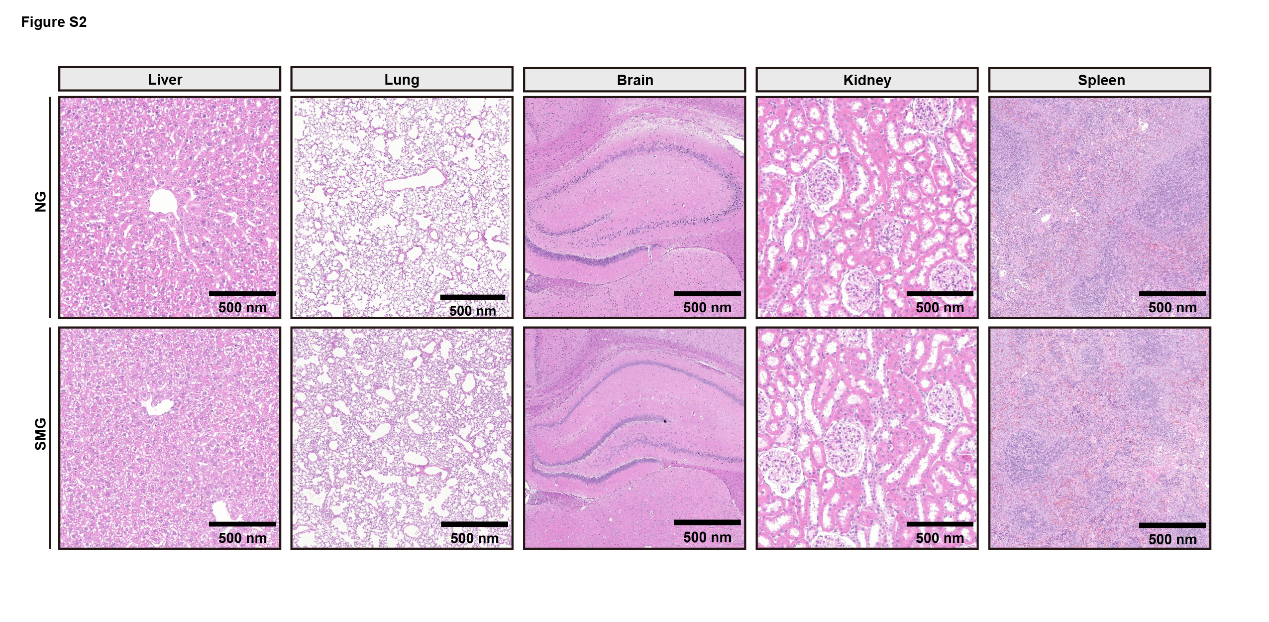
**

**Figure S2. Hematoxylin-Eosin (HE) staining of various organs in NG and SMG (scale bars, 500 nm).**


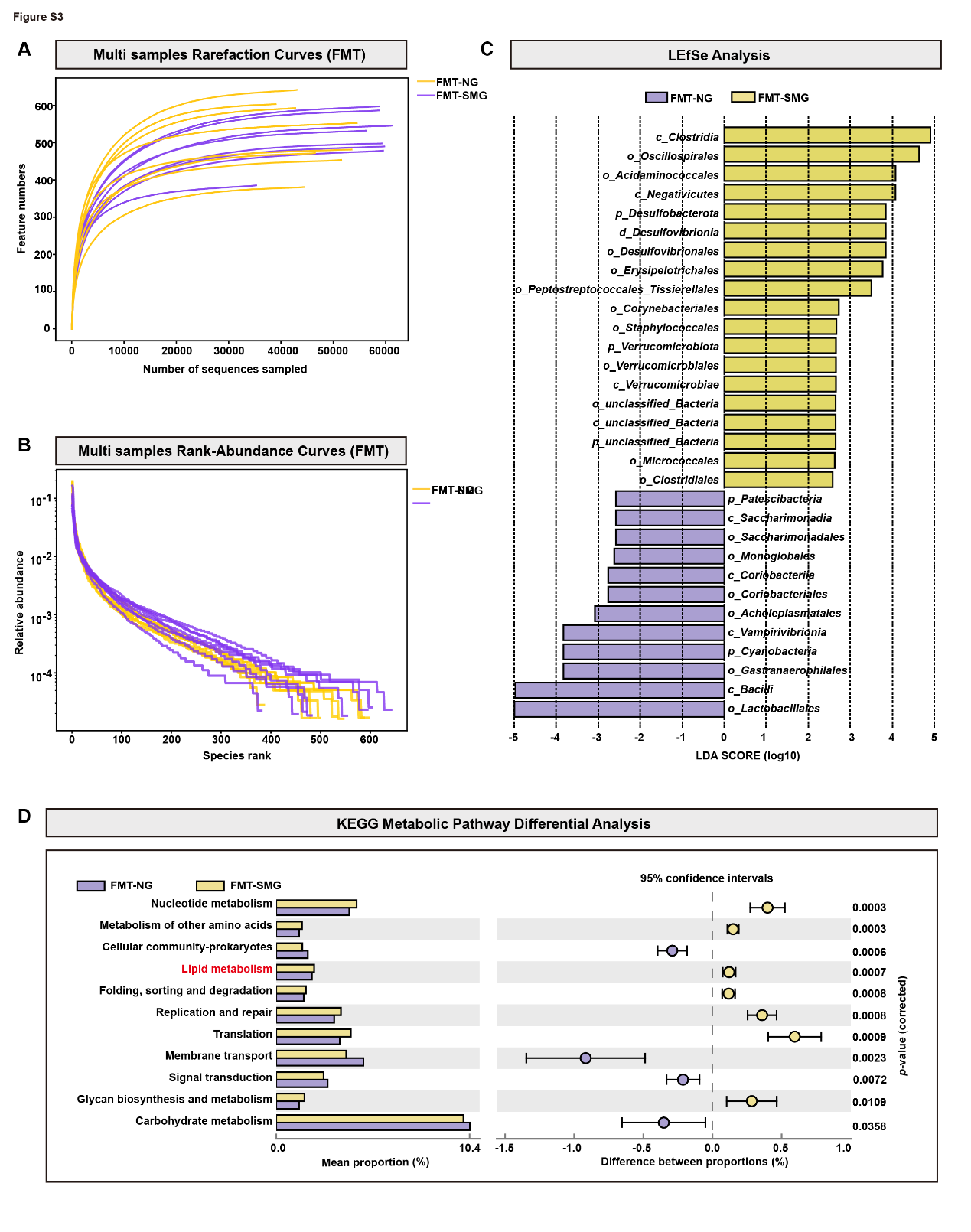


**Figure S3. Fecal Microbiota Transplantation (FMT) significantly modifies the composition of gut microbiota in rats.**

1. Multi samples rarefaction curves of 16S rRNA sequencing (n = 6).
2. Multi samples rank-abundance curves of 16S rRNA sequencing (n = 6).
3. Linear discriminative analysis (LDA) score of differentially enriched bacterial genera obtained from LEfSe analysis between the FMT-NG group and the FMT-SMG group. A genus with Kruskal-Wallis ≤ 0.05, as well as LDA ≥ 2, is shown.
4. Kyoto Encyclopedia of Genes and Genomes (KEGG) analysis evaluated the enriched pathways for the representative profiles of the FMT-NG group and the FMT-SMG group.


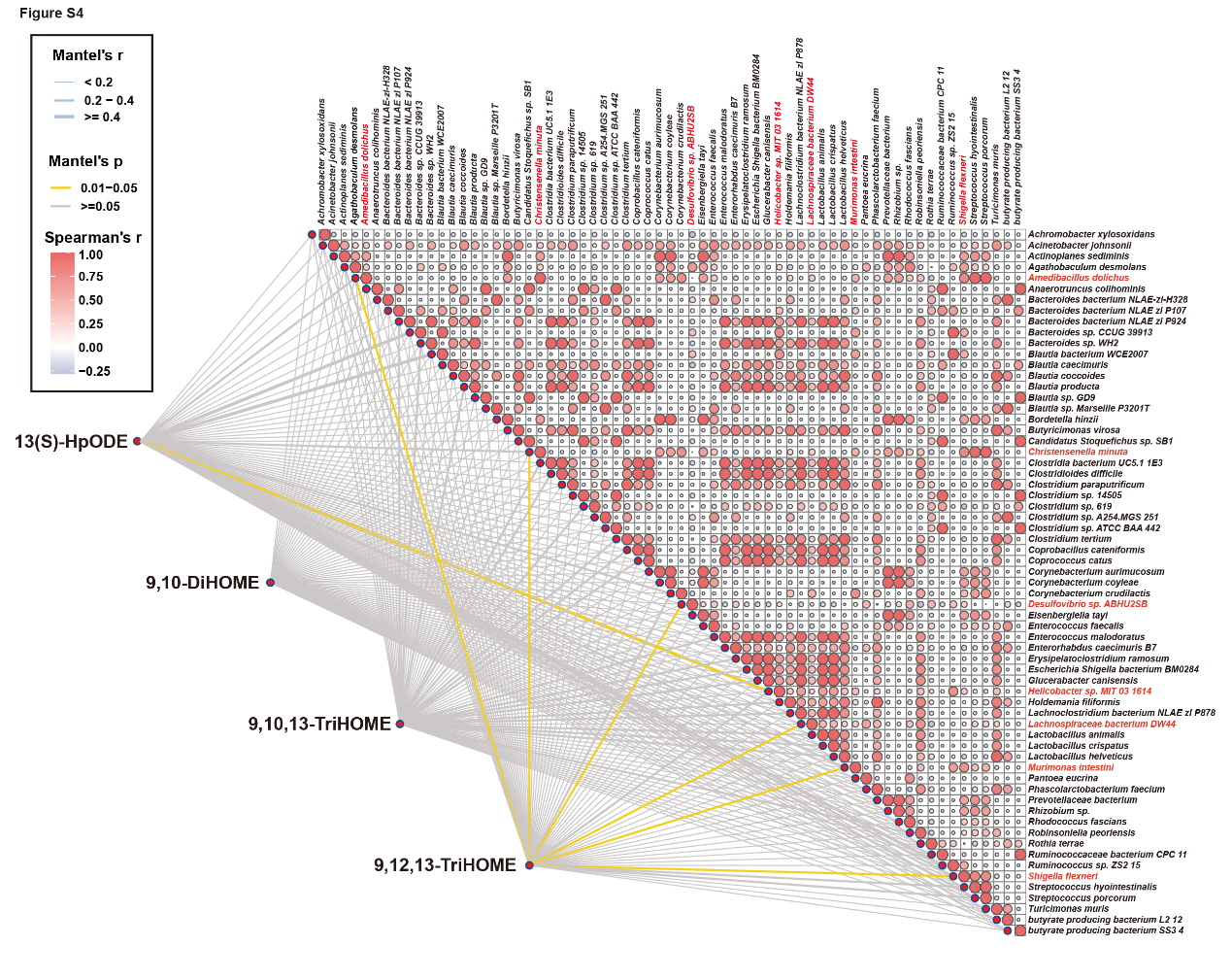


**Figure S4. Spearman’s correlation analysis of microbiome (bacterial species with increased abundance in SMG) and metabolome.**


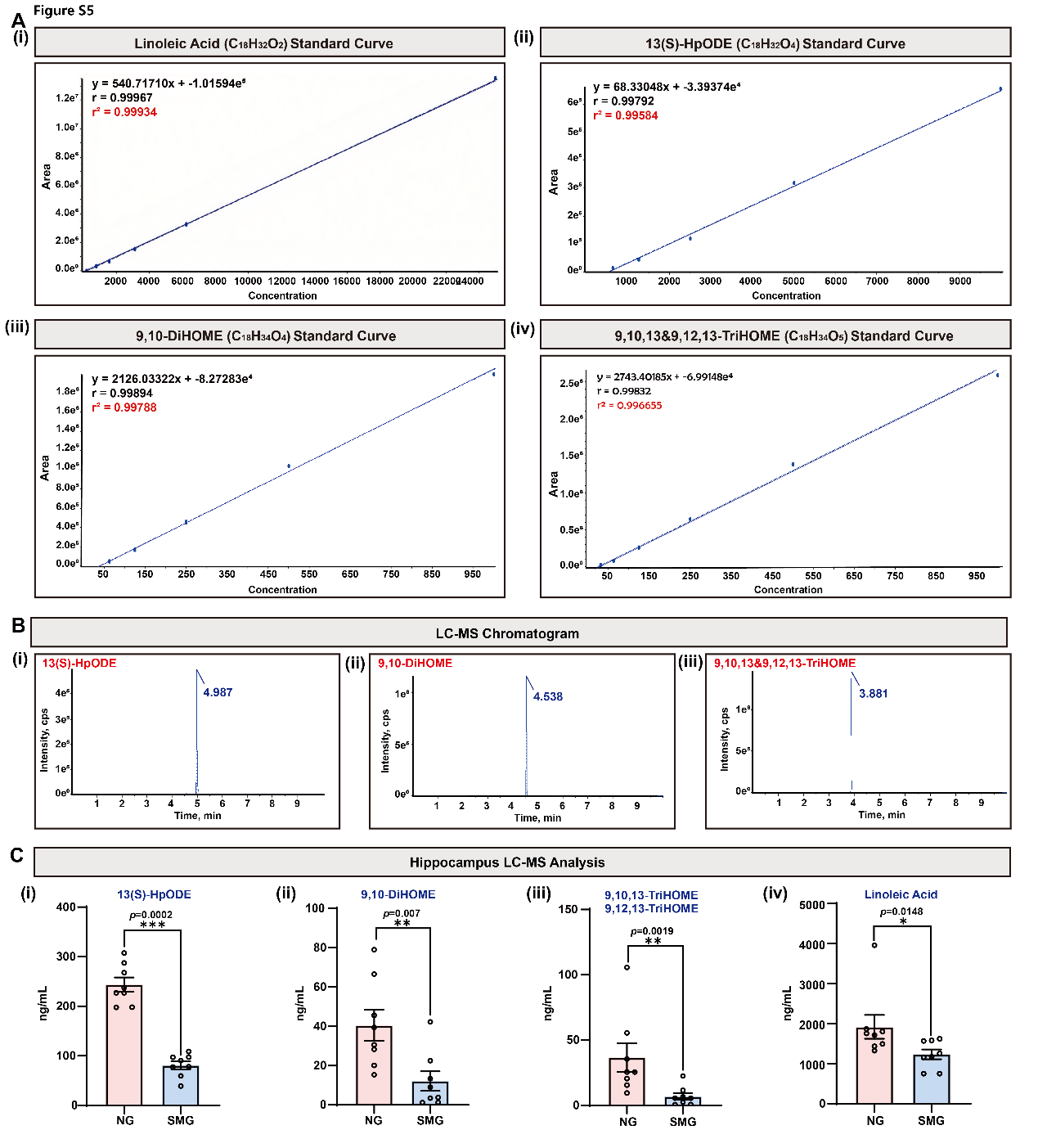


**Figure S5. Target LC-MS analysis of LA and its derivatives.**

1. Standard curves of (i) LA, (ii) 13(S)-HpODE, (iii) 9,10-DiHOME, and (iv) TriHOMEs (9,10,13-, and 9,12,13-).
2. LC-MS chromatograms of (i) 13(S)-HpODE, (ii) 9,10-DiHOME, and (iii) TriHOMEs (9,10,13-, and 9,12,13-). The LC-MS chromatogram of LA was shown in Figure 5B.
3. LC-MS analysis of LA and its derivative levels in hippocampus of NG/SMG groups (n = 8). (i) 13(S)-HpODE, *p* = 0.0002, (ii) 9,10-DiHOME, *p* = 0.007, (iii) TriHOMEs (9,10,13-, and 9,12,13-), *p* = 0.0019, and (iv) LA, *p* = 0.0148.

**
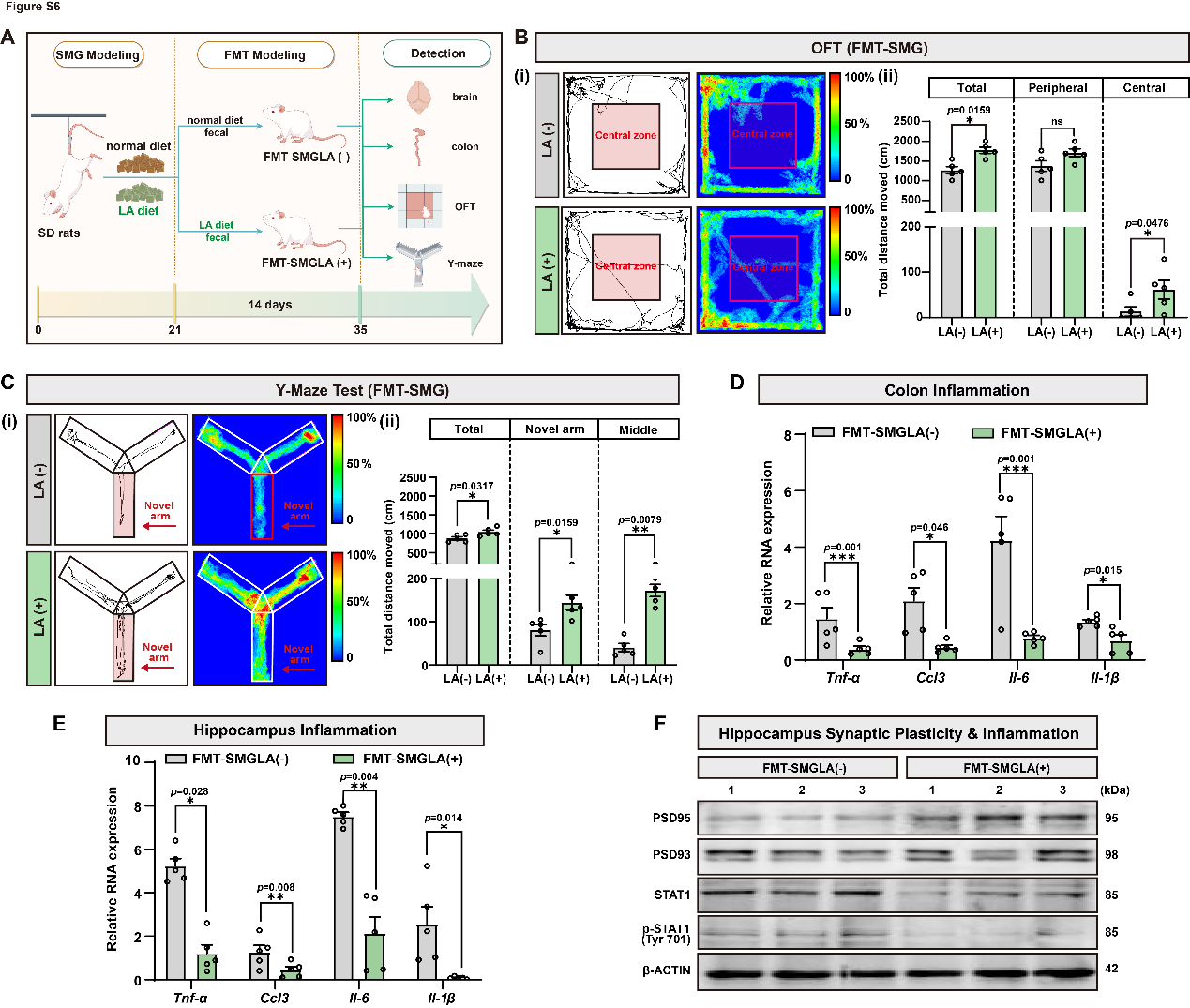
**

**Figure S6. LA-supplemented FMT-SMG alleviates brain dysfunction and neuroinflammation.**

1. Experimental scheme depicting fecal microbiome transplantation (FMT) from SMG (normal diet) or SMG-LA (+) (LA-supplemented diet) pre-treated rat donors to recipients. OFT and Y-maze behavioral tests were conducted on day 35.
2. OFT behavioral testing in the FMT-SMGLA (-) and FMT-SMGLA (+) groups. (i) Movement trajectories of rats in OFT; (ii) Distance moved in the total path (*p* = 0.0159), peripheral zone (ns), and central zone (*p* = 0.0476) in OFT.
3. Y-maze behavioral testing in the FMT-SMGLA (-) and FMT-SMGLA (+) groups. (i) Movement trajectories of rats in Y-maze; (ii) Distance moved in total path (*p* = 0.0317), novel arm (*p* = 0.0159) and middle zone (*p* = 0.0079).
4. qRT-PCR analysis of inflammation-related cytokines in the colons (n = 5). *Tnf-α*, *p* = 0.001; *Ccl3*, *p* = 0.046; *Il-6*, *p* =0.001; *Il-1β*, *p* = 0.015. Target gene mRNA is normalized to *β-actin* mRNA.
5. qRT-PCR analysis of inflammation-related cytokines in the hippocampus (n = 5). *Tnf-α*, *p* = 0.028; *Ccl3*, *p* = 0.008; *Il-6*, *p* =0.004; *Il-1β*, *p* = 0.014. Target gene mRNA is normalized to *β-actin* mRNA.
6. Immunoblot analysis of three independent individuals for PSD93/95, STAT1, and p-STAT1 (Tyr 701) in the hippocampus.

**
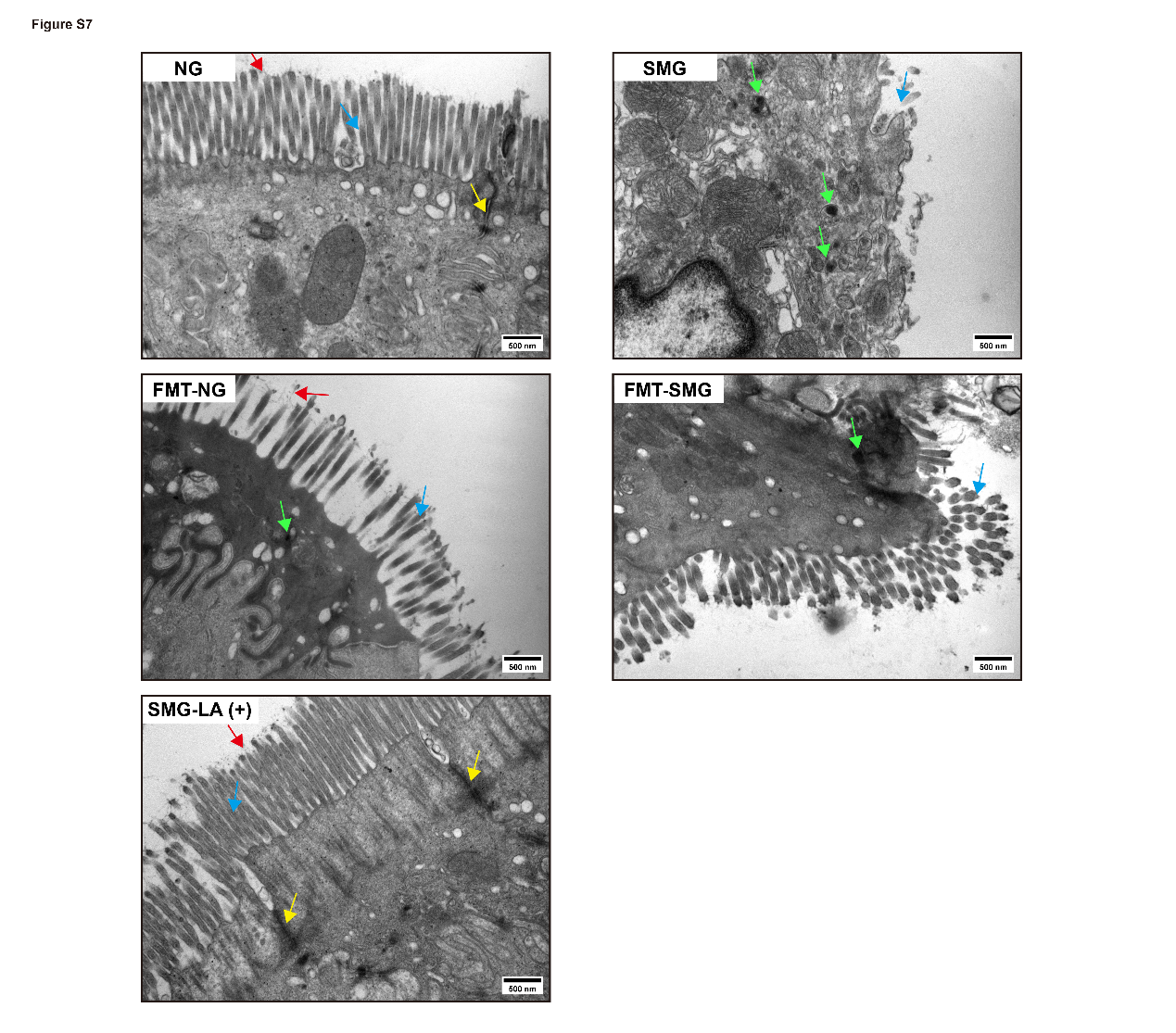
**

**Figure S7. Transmission electron microscopy of the colons (scale bars, 500 nm).**

**(i)** NG; **(ii)** SMG; **(iii)** FMT-NG; **(iv)** FMT-SMG; **(v)** SMG-LA (+). (Blue arrows: intact columnar epithelial cells; red arrows: complete glycocalyx; yellow arrows: tight junctions; green arrows: enlarged lysosomes).


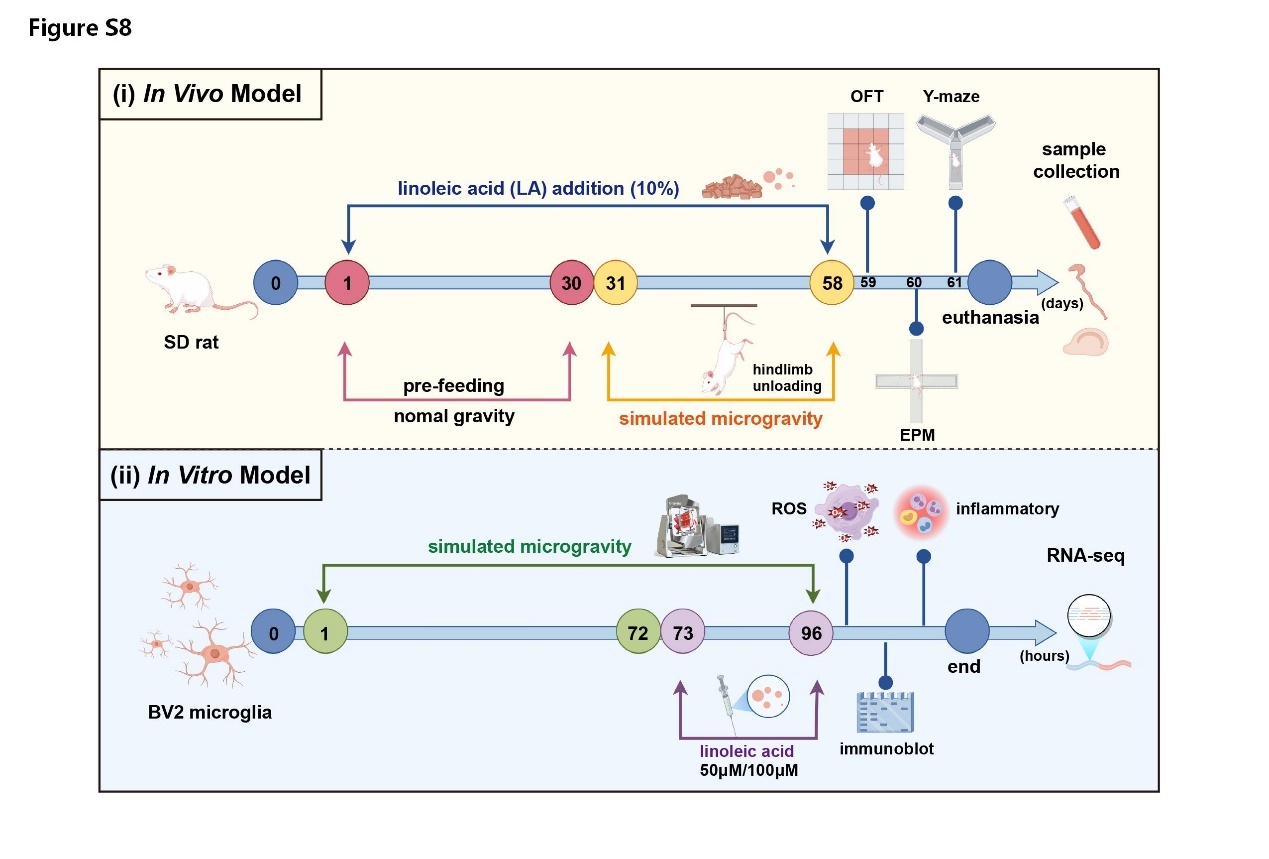


**Figure S8. Experimental design of LA intervention under SMG.**

**(i)** *In Vivo* Model: SD rats (n =5), pre-fed with a 10% LA-supplemented diet, underwent hindlimb unloading to simulate microgravity. Behavioral assessments (OFT, EPM, Y-maze) were performed at day 59 to 61, followed by tissue (blood, colon, and hippocampus) collection.

**(ii)** *In Vitro* Model: BV2 microglia were cultured under simulated microgravity and treated with LA (24 h). Samples were collected at 96 h for RNA sequencing, immunoblotting, and ROS measurement.


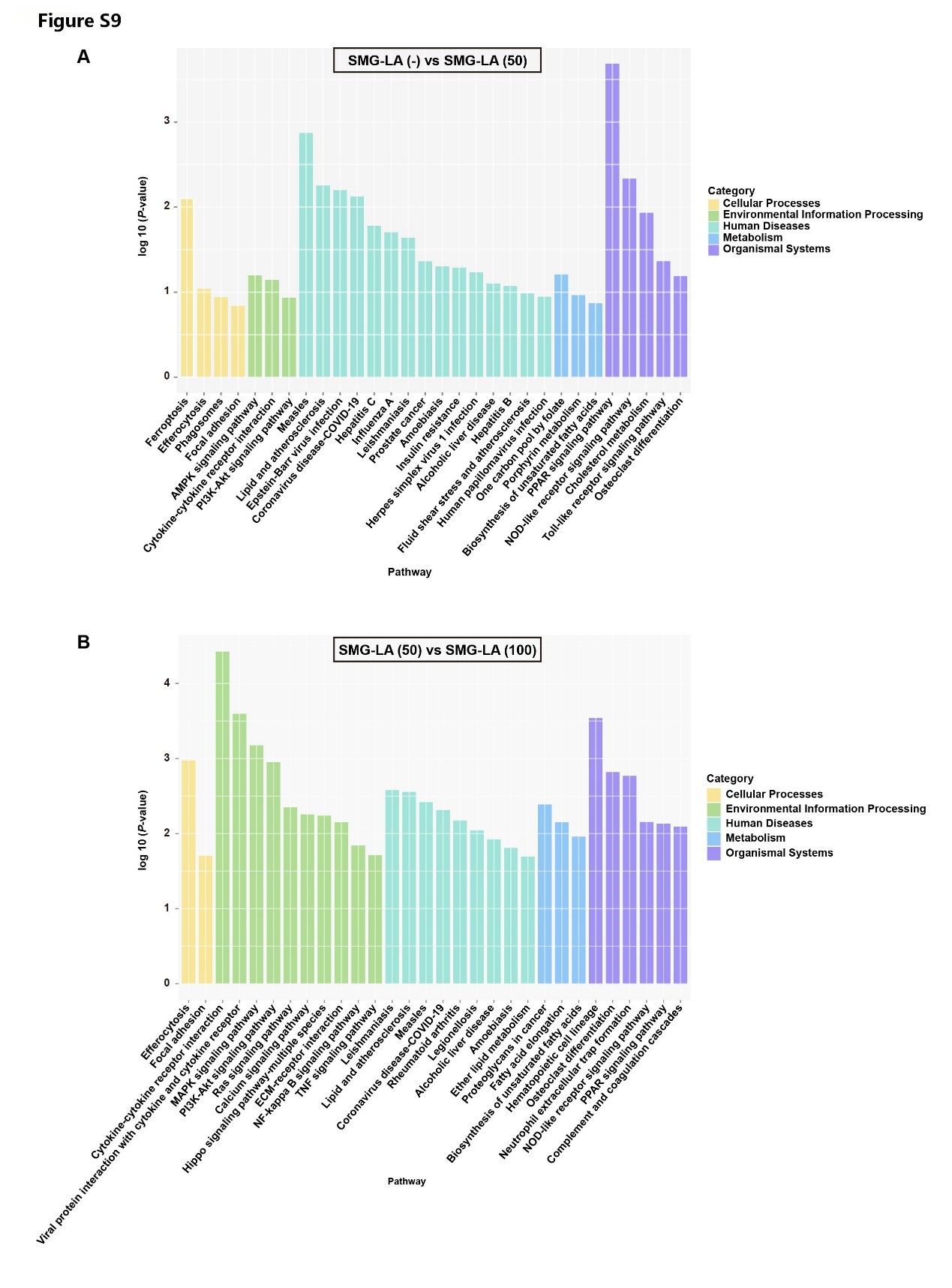


**Figure S9. KEGG pathway enrichment analysis bar plot (FDR < 0.05).**

**A.** SMG-LA (-) vs SMG-LA (50).

**B.** SMG-LA (50) vs SMG-LA (100).


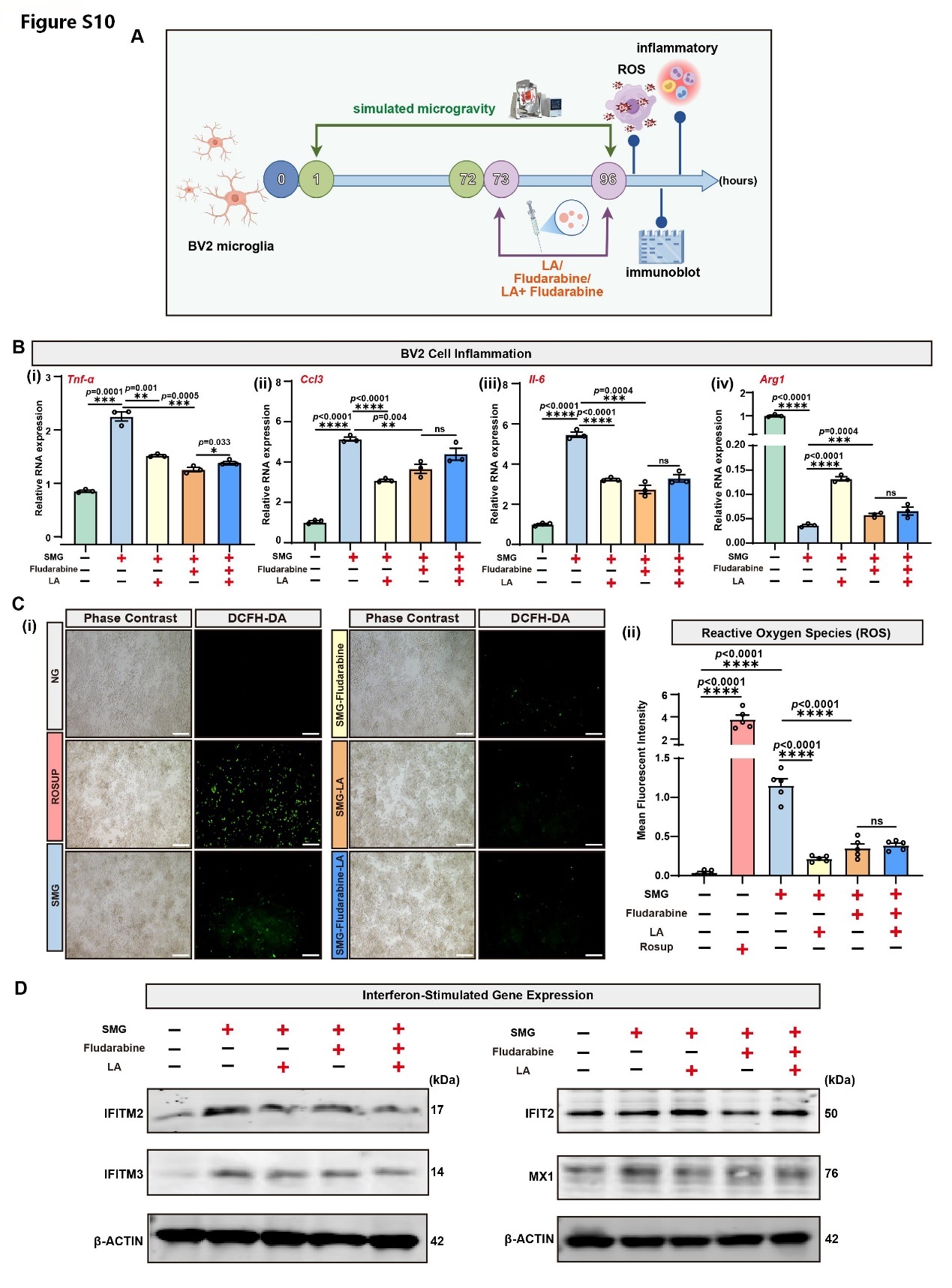


**Figure S10. LA inhibits microglial inflammatory activation depending on STAT1.**

**A.** Schematic of experimental procedure. BV2 microglia were subjected to SMG treatment, and experiments were performed at the time points of 0, 1, 72, and 96 h. LA, Fludarabine, or LA+Fludarabine was administered at 73 h, and inflammatory ROS and immunoblotting were detected at 96 hours.

**B.** Relative mRNA expression analysis of inflammation-related genes **(i) *Tnf-α*** (line 1/line 2, *p* = 0.0001; line 2/line 3, *p* = 0.001; line 2/line 4, *p* = 0.0005; line 4/line 5, *p* = 0.033), **(ii) *Ccl3*** (line 1/line 2, *p* < 0.0001; line 2/line 3, *p* < 0.0001; line 2/line 4, *p* = 0.004; line 4/line 5, ns), **(iii) *Il-6*** (line 1/line 2, *p* < 0.0001; line 2/line 3, *p* < 0.0001; line 2/line 4, *p* = 0.0004; line 4/line 5, ns), and **(iv) *Arg1*** (line 1/line 2, *p* < 0.0001; line 2/line 3, *p* < 0.0001; line 2/line 4, *p* = 0.0004; line 4/line 5, ns) in BV2 cells among different treatment groups.

**C.** ROS detection. **(i)** Phase Contrast microscopy and DCFH-DA fluorescent staining images of each group; **(ii)** Statistical analysis of mean ROS fluorescence intensity (line 1/line 2, *p* < 0.0001; line 1/line 3, *p* < 0.0001; line 3/line 4, *p* < 0.0001; line 3/line 5, *p* < 0.0001; line 5/line 6, ns).

**D.** Expression of interferon-stimulated genes IFITM2, IFITM3, IFIT2 and MX1 among different groups.
